# Concurrent Formation of Carbon–Carbon Bonds and Functionalized Graphene by Oxidative Carbon-Hydrogen Coupling Reaction

**DOI:** 10.1038/srep25824

**Published:** 2016-05-16

**Authors:** Kumika Morioku, Naoki Morimoto, Yasuo Takeuchi, Yuta Nishina

**Affiliations:** 1 Research Core for Interdisciplinary Sciences, Okayama University, Tsushimanaka, Kita-ku, Okayama 700-8530, Japan; 2 Graduate School of Medicine, Dentistry, and Pharmaceutical Sciences, Division of Pharmaceutical Sciences, Okayama University, Tsushimanaka, Kita-ku, Okayama 700-8530, Japan; 3 Precursory Research for Embryonic Science and Technology, Japan Science and Technology Agency, 4-1-8 Honcho, Kawaguchi, Saitama 332-0012, Japan

## Abstract

Oxidative C–H coupling reactions were conducted using graphene oxide (GO) as an oxidant. GO showed high selectivity compared with commonly used oxidants such as (diacetoxyiodo) benzene and 2,3-dichloro-5,6-dicyano-*p*-benzoquinone. A mechanistic study revealed that radical species contributed to the reaction. After the oxidative coupling reaction, GO was reduced to form a material that shows electron conductivity and high specific capacitance. Therefore, this system could concurrently achieve two important reactions: C–C bond formation *via* C–H transformation and production of functionalized graphene.

The carbon–hydrogen (C–H) bond is one of the most common chemical bonds in organic compounds. The C–H bonds of simple molecules may be cleaved to construct new carbon–carbon (C–C) bonds to obtain more sophisticated value-added chemicals^1^,^2^,^3^. C–H bonds are thermodynamically stable and kinetically inert, so activation of C–H bonds requires addition of a stoichiometric amount of a metal complex^4^,^5^,^6^,^7^,^8^ or catalytic amount of a metal complex combined with a stoichiometric amount of oxidant^9^,^10^,^11^,^12^. Recently, metal-free C–H transformation reactions have been achieved using stoichiometric amounts of hypervalent iodine compounds^13^,^14^,^15^,^16^, peroxides^17^,^18^, and quinone derivatives^19^,^20^. However, all of these reactions produce unwanted side products derived from the oxidant.

To achieve more efficient oxidative C–H coupling, we focused on graphene oxide (GO), which is produced from graphite *via* oxidation and exfoliation using KMnO_4_ in H_2_SO_4_ (Hummers’ method). In this process, numerous carbon atoms are transformed to sp^3^ hybridized C–O bonds (hydroxyl and epoxy groups) and C=O bonds (carbonyl and carboxyl groups). Because of its two-dimensional sheet structure, GO can be used to produce graphene by reduction of sp^3^ hybridized C–O bonds to recover sp^2^ hybridized carbons. GO is readily reduced in the presence of metals, alcohols, amines, proteins, and microorganisms, which means that GO has oxidizing ability^21^,^22^,^23^,^24^,^25^,^26^. Inspired by this, GO-promoted oxidation reactions have been investigated^27^,^28^,^29^,^30^,^31^,^32^; however, they have been limited to the oxidation of reactive alcohols, amines, and benzylic C–H bonds to date (Figs S1–S4). To improve the scope of GO as an oxidant, here we investigated additives that can promote C–H coupling reactions.

## Results and Discussion

**Figure 1:**
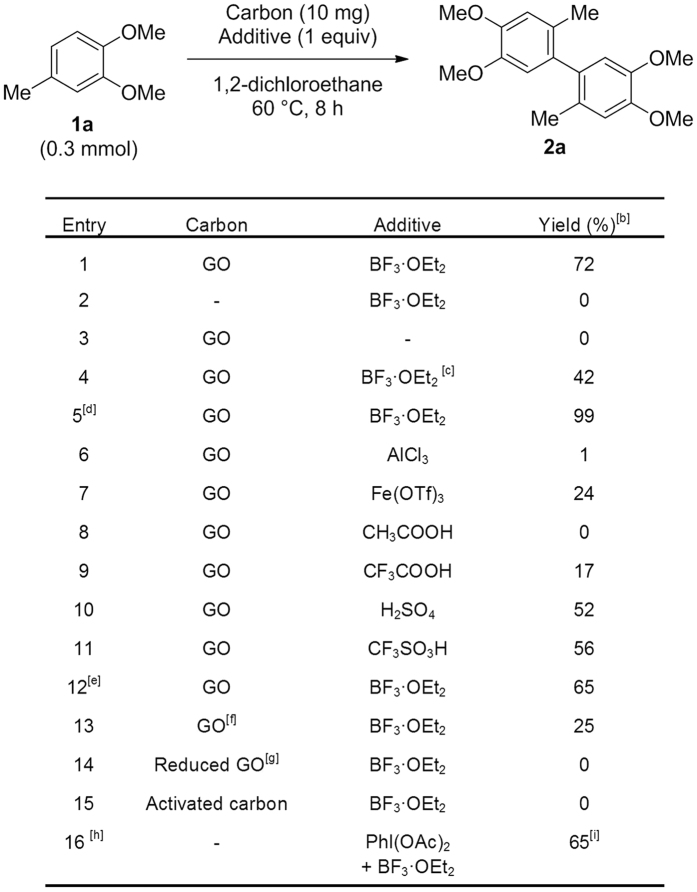
Survey of reaction conditions. ^[a]^(**a**) Reaction condition: **1a** (0.3 mmol), GO (oxygen content: 41.1 wt%, 10 mg), additive (0.3 mmol), 1,2-dichloroethane (0.2 mL) under Ar atomospher. (**b**) GC yield. (**c**) BF_3_·OEt_2_ (0.2 equiv), 24 h. (**d**) 20 mg of GO (oxygen content: 41.1 wt%) and 0.5 mL of 1,2-dichloroethane were used. (**e**) Reaction was performed under O_2_ atmosphere. (**f**) GO was prepared by Brodie’s method. (**g**) Reduced GO was prepared by reduction of GO with hydrazine. (**h**) Reaction condition: **1a** (0.3 mmol), GO (oxygen content: 41.1 wt%, 10 mg), PhI(OAc)_2_ (0.3 mmol), BF_3_·OEt_2_ (0.3 mmol), 1,2-dichloroethane (0.2 mL) under Ar atmosphere. (**i**) Acetoxylated products of **1a** were also formed.

Screening of various additives (Figs S5–S8) revealed that the oxidative C–H homocoupling reaction of 3,4-dimethoxytoluene (**1a**) proceeded using a combination of GO (41.1 wt% O) and boron trifluoride diethyl etherate (BF_3_·OEt_2_) (Fig. 1, Entry 1). The reaction did not proceed without GO or BF_3_·OEt_2_ (Fig. 1, Entry 2 and 3). Although BF_3_·OEt_2_ worked as a catalyst (Fig. 1, Entry 4), the reaction was slow because of inhibition by water produced during the course of the reaction (Fig. S9). The reaction proceeded quantitatively when the amount of GO was increased (Fig. 1, Entry 5). Metallic Lewis acids AlCl_3_ and Fe(OTf)_3_ were not effective in the reaction (Fig. 1, Entry 6 and 7), while Brønsted acids promoted the reaction. The pK_a_ of each Brønsted acid was correlated with the product yield. A lower pK_a_ gave a higher yield; CH_3_COOH (pK_a_ = 4.75, 0% yield), CF_3_COOH (pK_a_ = 0.23, 17% yield), H_2_SO_4_ (pK_a_ = −3.0, 52% yield), and CF_3_SO_3_H (pK_a_ = −12, 56% yield) (Fig. 1, Entry 8–11). The reaction was not affected by oxygen (Fig. 1, Entry 12) or other oxidants (Table S4). GO prepared by Brodie’s method^33^,^34^,^35^,^36^ gave the product in low yield (Fig. 1, Entry 13). Reduced GO (rGO) and activated carbon did not promote the reaction (Fig. 1, Entry 14 and 15), suggesting that the specific oxygen functional groups of GO contributed to it. For comparison, we employed a hypervalent iodine compound (PhI(OAc)_2_), which is often used in metal-free oxidative coupling reactions, for this transformation. The reaction proceeded in 65% yield; however, unidentified acetoxylated compounds were also obtained as byproducts (Fig. 1, Entry 16). Therefore, GO was found to be a selective oxidant for the oxidative C–H coupling reaction.

**Figure 2:**
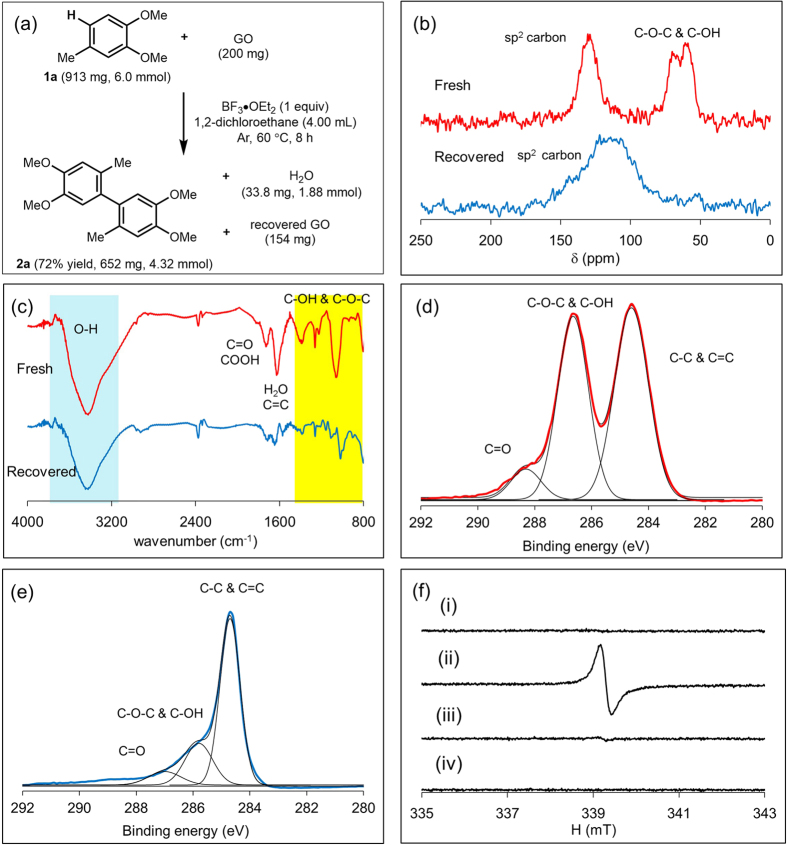
Comparison of fresh and recovered GO samples from oxidative C–H coupling. (**a**) Mass balance of the reaction, and characterization of fresh GO (oxygen content: 41.1 wt%), and recovered GO after the oxidative C–H coupling reaction; (**b**) solid state ^13^C NMR spectra, (**c**) IR spectra, (**d**) XPS C1s region of fresh GO, and (**e**) XPS C1s region of recovered GO. (**f**) ESR spectra of (i) GO (O: 50.7 wt%), (ii) GO with 3,4-dimethoxytoluene and BF_3_·OEt_2_, (iii) GO with 3,4-dimethoxytoluene, and (iv) GO with BF_3_·OEt_2_.

To further clarify the role of GO, the stoichiometry of the reaction was analyzed (Fig. 2a). We reacted **1a** (6.00 mmol) and GO (41.1 wt% O, 200 mg) under the conditions described for Entry 1 of Fig. 1. The reaction, which proceeded in 72% yield, was regarded as the removal of 4.32 mmol of hydrogen atoms from **1a**, and the weight of recovered GO was 154 mg. Elemental analysis (Table S2) indicated that the carbon content of GO before the reaction was 110 mg (55.0 wt% of 200 mg), and that of recovered GO was 114 mg (74.0 wt% of 154 mg). The hydrogen content of GO before and after the reaction was 4.1 mg, so hydrogenation of GO did not occur. The slight increase in the carbon content of GO could be derived from the adsorption of **1a** and/or **2a** *via* π–π interactions. However, we assume that such adsorption is negligible because of the little change in hydrogen content and high mass balance of **1a** and **2a**. These results suggest that oxygen is exclusively eliminated from GO during the reaction. Supposing that all of the weight loss of GO during the reaction (46 mg) is derived from the elimination of oxygen, 2.88 mmol of oxygen contributed to the oxidation. Based on these observations, one oxygen atom of GO abstracts one or two hydrogen atoms from **1a**. To identify the oxygen functional groups involved in the reaction, structural analyses of fresh and recovered GO samples were performed by solid-state ^13^C nuclear magnetic resonance (NMR) spectroscopy (Fig. 2b), Fourier transform infrared (FT-IR) spectroscopy (Fig. 2c), and X-ray photoelectron spectroscopy (XPS) (Fig. 2d,e). The solid-state ^13^C NMR spectrum of fresh GO showed three peaks at 60, 70 and 130 ppm (Fig. 2b red line). Meanwhile, the solid-state ^13^C NMR spectrum of recovered GO (Fig. 2b blue line) revealed a considerable decrease in the content of C–O bonds (60–70 ppm), along with a broad resonance peak at 100–130 ppm. These spectral changes are consistent with the generation of sp^2^ hybridized carbons accompanied by the loss of oxygen functional groups. The peaks at around 60 and 70 ppm are attributed to hydroxyl and epoxy groups, respectively^37^,^38^. However, ^13^C NMR spectra of model compounds (Fig. S10) suggested that both peaks could originate from epoxy groups. FT-IR spectra of recovered GO also showed the remarkable decrease in the content of oxygen functional groups at 800–1200 cm^−1^. Unfortunately, it was difficult to identify the actual functional groups because of the complexity of the fingerprint region. There was no marked change in the C–H and O–H stretching vibration peaks at 2800–3600 cm^−1^, suggesting that C–H bonds are not formed and O–H bonds are not eliminated (Fig. 2c, blue line). XPS analysis of the C 1s region of fresh GO confirmed that the main oxygen functional groups were C–O bonds (Fig. 2d). The decrease in the content of C–O bonds induced by the oxidative coupling reaction was further evidenced by XPS measurement of the recovered GO (Fig. 2e). Considering the above-mentioned stoichiometry calculation and structural analyses, we believe that epoxy groups were the main oxygen functional group of GO^39^,^40^,^41^,^42^ and acted as an oxidant to form H_2_O. The formation of water during the reaction was confirmed by the Karl–Fischer method (Table S5).

To obtain mechanistic insights into the reaction^41^,^42^, the oxidative C–H coupling reaction with GO was performed in the presence of radical scavenger 2,2,6,6-tetramethylpiperidine-1-oxyl (TEMPO). TEMPO prevented the reaction from proceeding (see ESI). Furthermore, a kinetic isotope effect was not observed when 6-deuterated **1a** was used (see ESI). These results suggest the participation of radical species that can promote rapid hydrogen abstraction from **1a**. To prove the formation of radical species *in situ*, we performed electron spin resonance (ESR) analysis. The ESR spectrum of highly oxidized GO (oxygen content: 50.7 wt%) did not contain a signal (Fig. 2f (i)). When **1a** and BF_3_·OEt_2_ were added to GO, an ESR signal appeared (Fig. 2f (ii)), whereas negligible signal was observed when only **1a** or BF_3_·OEt_2_ was added (Fig. 2f (iii) and (iv)). The ESR signal generated by the reaction of **1a**, GO, and BF_3_·OEt_2_ is consistent with the so-called “carbon radical” stabilized by a resonance effect. A different ESR signal derived from radical cations was observed when **1a**, PhI(OAc)_2_, and BF_3_·OEt_2_ were mixed^43^.

The interaction between GO, **1a**, and BF_3_·OEt_2_ was further investigated. The FT-IR spectrum of **1a** did not change upon addition of BF_3_·OEt_2_, while that of GO shifted upon addition of BF_3_·OEt_2_ (Fig. S11). ^1^H NMR spectra showed the same tendency; the signal from the ethyl group of BF_3_·OEt_2_ shifted when GO was added, while no change occurred when **1a** was added (Fig. S12). These observations suggest that BF_3_·OEt_2_ activates GO to form radical species.

**Figure 3:**
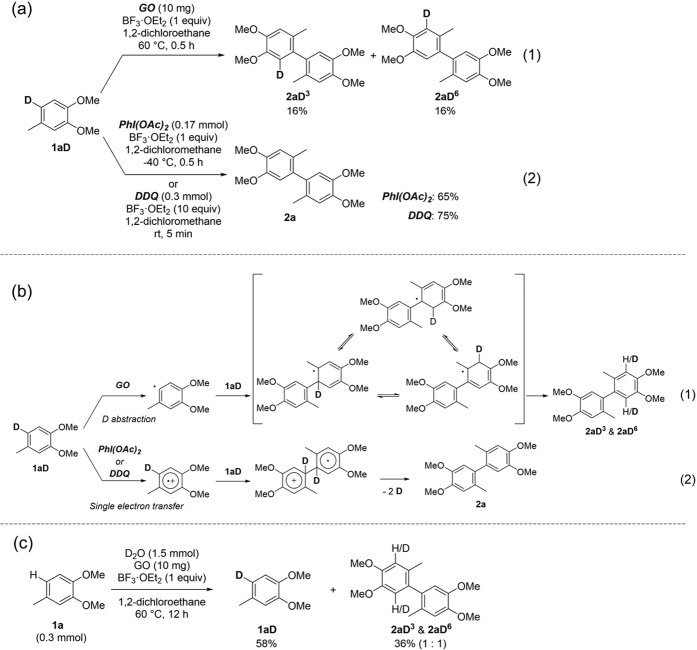
Mechanistic investigation. (**a**) Deuterium labelling experiment; (1) GO promoted the formation of **2aD**^**3**^ and **2aD**^**6**^ (both in 16% yield). (2) Phl(OAc)_2_ and DDQ promoted the formation of only **2a** in 65% and 75% yield, respectively. (**b**) Proposed reaction mechanisms promoted by (1) GO, (2) Phl(OAc)_2_ or DDQ. (**c**) H/D exchange experiment with D_2_O. Only **1aD, 2aD**^**3**^, and **2aD**^**6**^ were observed in yields of 58%, 18%, and 18%, respectively.

The principal difference between GO and other organic oxidants was determined by deuterium labeling experiments. When 6-deutero-3,4-dimethoxytoluene (**1aD**) was used, GO promoted the scrambling of D/H to form **2aD**^**3**^ and **2aD**^**6**^ (Fig. 3a-1); in contrast, PhI(OAc)_2_ and 2,3-dichloro-5,6-dicyano-*p*-benzoquinone (DDQ) produced only **2a** (Fig. 3a-2). The proposed reaction mechanisms with GO, PhI(OAc)_2_ and DDQ are presented in Fig. 3b. When GO is used, deuterium abstraction of **1aD** could occur to form an aryl radical, which readily reacts with another **1aD**. The formed 3° radical is relatively stable, so scrambling of H/D could occur before elimination of hydrogen or deuterium (Fig. 3b-1). In contrast, PhI(OAc)_2_ and DDQ are proposed to produce a radical cation *via* single electron transfer, which was detected by ESR (Fig. 3b-2)^20^,^43^. The formation of an aryl radical by GO was proved by deuterium exchange reaction in the presence of D_2_O. The oxidative coupling reaction proceeded in 36% yield to form **2aD**^**3**^ and **2aD**^**6**^ without **2a**, and **1a** was deuterated to form **1aD** (Fig. 3c). For comparison, the H/D exchange was investigated using PhI(OAc)_2_; however, no deuterium was incorporated in **1a** and **2a** in this case. These results show that the oxidative coupling reactions using GO and PhI(OAc)_2_ as the oxidant could pass through different intermediates.

**Figure 4:**
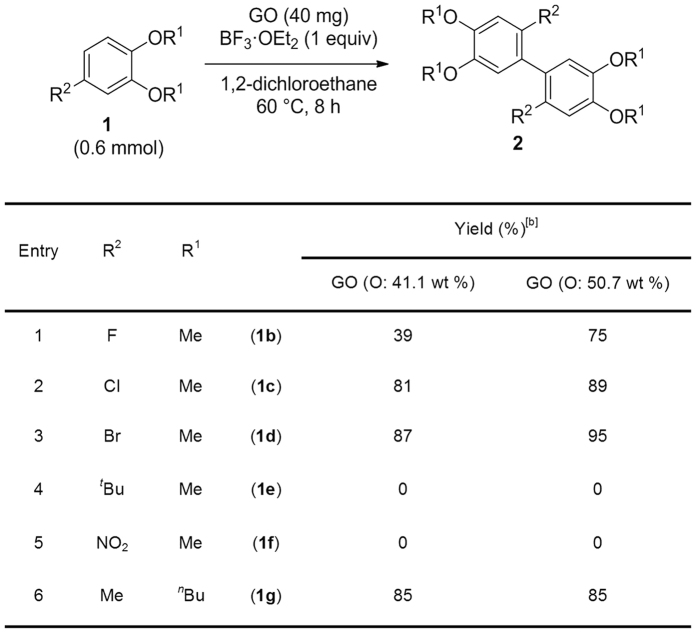
Substrate scope. ^[a]^(**a**) Reaction condition: **1** (0.6 mmol), GO (40 mg), BF_3_·OEt_2_ (0.6 mmol), 1,2-dichloroethane (1.0 mL) under Ar atmosphere. (**b**) Isolated yield.

**Figure 5:**
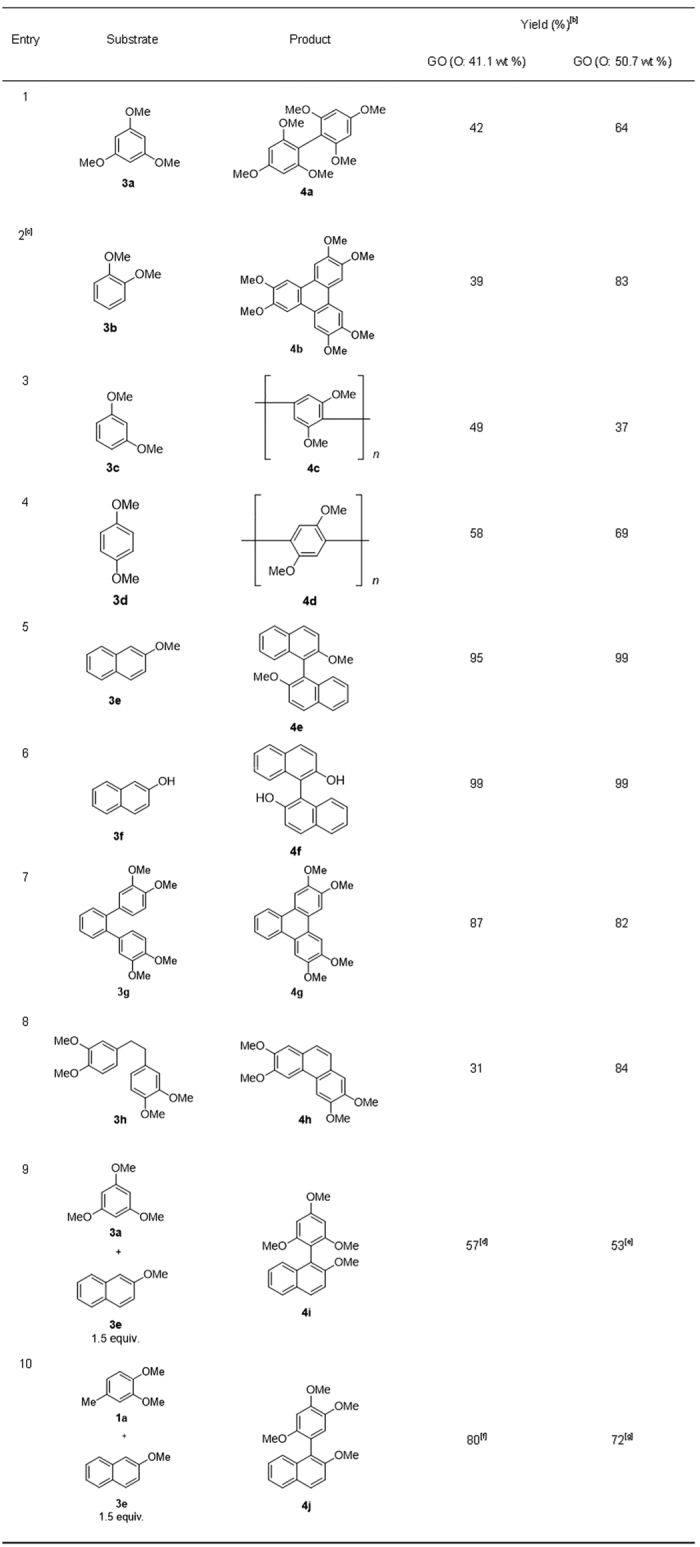
Substrate scope with various aromatic compounds. ^[a]^(**a**) Reaction condition: **1** (0.6 mmol), GO (40 mg), BF_3_·OEt_2_ (0.6 mmol), 1,2-dichloroethane (1.0 mL) under Ar atmosphere. (**b**) Isolated yield. (**c**) Reaction condition: 1,2-dimethoxybenzene (0.6 mmol), GO (100 mg), BF_3_·OEt_2_ (2.0 mL) under Ar atmosphere, 60 °C, 8 h. (**d**) Homocoupling product of **3a** and **3e** were produced in 1% and 11% yield. (**e**) Homocoupling product of **3a** and **3e** were produced in 1% and 6% yield. (**f**) Homocoupling product of **3a** and **3e** were produced in 19% and 29% yield. (**g**) Homocoupling product of **1a** and **3e** were produced in 19% and 31% yield.

Next, the scope of the reaction was investigated using aromatic compounds with a variety of functional groups (Fig. 4). When halogenated aromatic compounds bearing a fluoro, chloro, or bromo group were used as starting materials, the product yield decreased as the electronegativity of the substituent increased (Fig. 4, Entry 1–3). A bulky or electron-withdrawing group prevented the reaction (Fig. 4, Entry 4, 5), while longer alkoxy groups did not affect the reaction (Fig. 4, Entry 6). Figure 5 shows the reaction scope for various aromatic compounds. The reaction proceeded in moderate yield when 1,3,5-trimethoxybenzene (**3a**) was used (Fig. 5, Entry 1). Trimerization occurred when 1,2-dimethoxybenzene (**3b**) was employed (Fig. 5, Entry 2). When another dimethoxybenzene (**3c** or **3d**) was used, oligomerization occurred (Fig. 5, Entry 3, 4) (for mass spectral analysis, see ESI). A naphthalene derivative (**3e** or **3f**) also underwent the oxidative coupling reaction to yield a binaphthyl compound (**4e** and **4f**) (Fig. 5, Entry 5, 6). Intramolecular oxidative coupling reaction of a terphenyl derivative (**3g**) also proceeded to give **4g** in good yield (Fig. 5, Entry 7). An anchored diphenyl compound (**3h**) underwent intramolecular oxidative coupling and successive dehydrogenative aromatization to give **4h** (Fig. 5, Entry 8). Cross-coupling reactions proceeded in moderate to good yields when **1a** or **2a** and **3e** were combined, although small amounts of undesired homocoupling products were detected (Fig. 5, Entry 9, 10). In some cases, highly oxidized GO (oxygen content: 50.7 wt%) showed higher reactivity than less oxidized GO (oxygen content: 41.1 wt%). (Fig. 4, Entry 2, 4, 6).

In general oxidation reactions, consumption of an oxidant is wasteful. rGO has great potential for practical use in conductive materials, supercapacitor electrodes, and sensors^44^,^45^,^46^,^47^. rGO has been produced by reduction with hydrazine^48^, sodium borohydride^49^, and alcohols^50^, and by hydrothermal^51^, solvothermal^52^ and thermal annealing^53^ reactions of GO. The reduction process to form rGO consumes a reductant or energy, and is an inefficient operation. Recovered GO from the oxidative C–H coupling reaction may behave as rGO, and the electrical conductivity and specific capacitance of the recovered GO could be controlled by changing the oxygen content of the original GO^54^. When GO with the oxygen content of 41.1 wt% was used in the reaction, the recovered GO showed high electron conductivity (40.0 S/cm), because graphene-like structure was readily recovered, however the surface area was low (24.1 m^2^/g). In contrast, highly oxidized GO with the oxygen content of 50.7 wt% was reduced in the course of the reaction to show high surface area (47.9 m^2^/g). Because of the high surface area and pseudocapacitance induced by redox reaction of the remained oxygen functional groups on GO, the recovered GO showed high specific capacitance (166 F/g). For comparison, the electron conductivity and specific capacitance of rGO produced by hydrazine reduction were 39.0 S/cm and 80.8 F/g, respectively (Table S6).

## Conclusion

Oxidative C–H coupling was performed using GO as an oxidant. Epoxy groups in the GO structure were activated by the acid additive to produce active radical species. The GO recovered after the oxidative C–H coupling reaction showed electrical conductivity, and has potential to work as a supercapacitor electrode. Therefore, this system could achieve two important reactions, C–H transformation and rGO production, in one pot.

## Methods

### Synthesis of graphene oxide

Graphite (3.0 g) was stirred in 95% H_2_SO_4_ (75 mL). The required amount of KMnO_4_ (6.0 and 15 g) was gradually added to the solution keeping the temperature <10 °C. The mixture was then stirred at 35 °C for 2 h. The resulting mixture was diluted by water (75 mL) under vigorous stirring and cooling so that temperature does not exceed 50 °C. The suspension was further treated by adding 30% aq. H_2_O_2_ (7.5 mL). The resulting graphite oxide suspension was purified by centrifugation with water until neutralization. Several GO were analyzed by CHNS elemental analysis to evaluate the oxygen content.

### Experimental procedure of mass balance of the reaction

To the solution of 1,2-dichloroethane (4.0 mL), GO (200 mg), 3,4-dimethoxytoluene (913 mg, 6.0 mmol) and BF_3_·OEt_2_ (852 mg, 6.0 mmol) were added under Ar atmosphere and stirred at 60 °C for 8 h. After the reaction, reaction mixture was filtrated. Recovered GO was dried under reduced pressure and CHNS elemental analysis was performed. The filtrate was analyzed by GC using *n*-decane as an internal standard Fig. 2a.

### ESR analysis

Spectrometer parameters: Microwave power: 1.0 mW. Microwave Freq.: 9.5 GHz. Scan time: 1 min. Modulation amplitude: 0.01 mT. Modulation Freq.: 100 kHz. Receiver gain: 16. Time constant: 0.01. ESR spectra was measured under ambient pressure at rt. 10.0 mg of GO was added to the quartz sample tube. Then each reactant (0.3 mmol) was added to the tube, and the measurement was performed Fig. 2f.

### Kinetic isotope effect

To the solution of 1,2-dichloroethane (0.2 mL), GO (10.0 mg), BF_3_·OEt_2_ (42.6 mg, 0.3 mmol) and 3,4-dimethoxytoluene (45.7 mg, 0.3 mmol) or 6-deuterio-3,4-dimethoxytoluene (46.0 mg, 0.3 mmol) were added under Ar atmosphere and stirred at 60 °C for 8 h. After the reaction, reaction mixture was quenched by AcOEt and water. The organic phase was concentrated under reduced pressure and purified by column chromatography. However unseparatable products were obtained when 3,4-dimethoxytoluene-*d* was used. Structures of product were determined by ^1^H NMR and EI-MS.

## Additional Information

**How to cite this article**: Morioku, K. *et al*. Concurrent Formation of Carbon-Carbon Bonds and Functionalized Graphene by Oxidative Carbon-Hydrogen Coupling Reaction. *Sci. Rep.* **6**, 25824; doi: 10.1038/srep25824 (2016).

## Supplementary Material

Supplementary Information
