# Supplementary material for: Concurrent Formation of Carbon–Carbon Bonds and Functionalized Graphene by Oxidative Carbon-Hydrogen Coupling Reaction
Source: Sci Rep. 2016 May 16;6:25824. doi: 10.1038/srep25824 (PMC4867571; doi:10.1038/srep25824)
Supplement: Supplementary Information [file srep25824-s1.doc]

Concurrent Formation of Carbon–Carbon Bonds and Functionalized Graphene by Oxidative Carbon-Hydrogen Coupling Reaction

Kumika Morioku^a^, Naoki Morimoto^b^, Yasuo Takeuchi^b^ and Yuta Nishina^a,c^*

^a^ Research Core for Interdisciplinary Sciences, Okayama University, Tsushimanaka, Kita-ku, Okayama 700-8530, Japan

^b^ Graduate School of Medicine, Dentistry, and Pharmaceutical Sciences, Division of Pharmaceutical Sciences, Okayama University, Tsushimanaka, Kita-ku, Okayama 700-8530, Japan

^c^ Precursory Research for Embryonic Science and Technology, Japan Science and Technology Agency, 4-1-8 Honcho, Kawaguchi, Saitama 332-0012, Japan

Fax: (+81) 86-251-8483

E-mail: nisina-y@cc.okayama-u.ac.jp

Homepage: http://www.tt.vbl.okayama-u.ac.jp/

**Supplementaly information**

1. **Materials.**

SP-1 Graphite was purchased from BAY CARBON Inc. Acetylene black (DENKA black) was provided by DENKI KAGAKU KOGYO Inc. and KF POLYMER 9130 was purchased from Kureha Co. All reagents were used directly without further purification.

**1e**^1^**, 1f**^2^**, 3g**^3^ and **3h**^4^ were synthesized by using known method.

**1c**, **1g** and **2aD** were synthesized below mentioned methods.

1. **General Information**

ESR spectra were measured by BRUKER ESP300E. Atomic absorption was made by using SHIMADZU AA-6300. XPS spectra were measured by SHIMADZU Kratos AXIS-ULTRA DLD with pass energy of 20 eV. The cyclic voltammograms of the second cycle were collected on SOLARTRON 1287 electrochemical instrument at the scan rate 20 mVs^-1^. The CV potential range was -0.8 to 0.1 V versus Hg / HgO in 1.0 M KOH electrolyte with a Pt foil as counter electrode.

1. **Preparation of several oxidation degree of GO**

Graphite (3.0 g) was stirred in 95% H_2_SO_4_ (75 mL). The required amount of KMnO_4_ (1.5, 3.0, 6.0, 9.0 and 15 g) was gradually added to the solution keeping the temperature <10 °C. The mixture was then stirred at 35 °C for 2 h. The resulting mixture was diluted by water (75 mL) under vigorous stirring and cooling so that temperature does not exceed 50 °C. The suspension was further treated by adding 30% aq. H_2_O_2_ (7.5 mL). The resulting graphite oxide suspension was purified by centrifugation with water until neutralization. Several GO were analyzed by CHNS elemental analysis to evaluate the oxygen content.

Table S1. Elemental compositions of several GO.


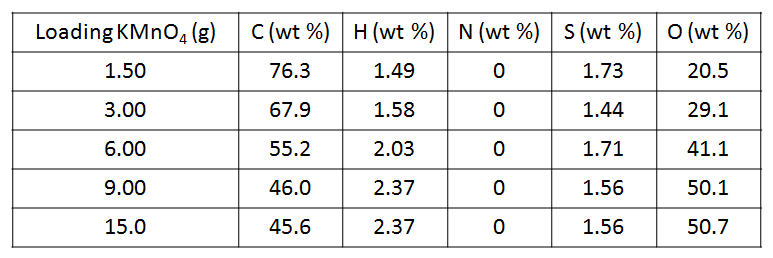


1. **Typical procedure for the optimization of reaction condition (Fig. 1)**

To the solution of 1,2-dichloroethane (0.2 mL), GO (10.0 mg), 3,4-dimethoxytoluene (45.7 mg, 0.3 mmol) and boron trifloride (42.6 mg, 0.3 mmol) were added under Ar atmosphere and stirred at 60 °C for 8 h. After the reaction, the reaction mixture was analyzed by GC using *n*-decane as an internal standard.

1. **Typical procedure for substrate scope (Fig. 4, 5)**

To the solution of 1,2-dichloroethane (0.5 mL), GO (40.0 mg), substrate (0.6 mmol) and BF_3_·OEt_2_ (85.2 mg, 0.6 mmol) were added under Ar atmosphere and stirred at 60 °C for 8 h. After the reaction, reaction mixture was quenched by AcOEt and water. The organic phase was concentrated under reduced pressure and purified by column chromatography.

1. **The result of elemental analysis for Fig. 2a.**

Table S2. Elemental compositions of GO and recovered GO.


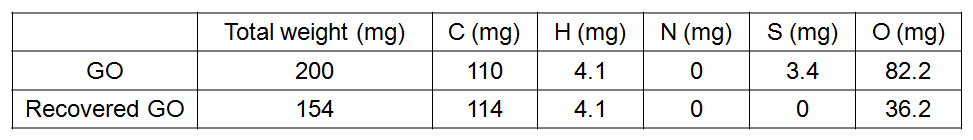


1. **Typical procedure for trimerization of 1,2-dimethoxybenzene (Fig. 5, Entry 2)**

To the solution of BF_3_·OEt_2_ (2.0 mL), GO (100 mg) and 1,2-dimethoxybenzene (82.9 mg, 0.6 mmol) were added under Ar atmosphere and stirred at 60 °C for 8 h. After the reaction, the reaction mixture was filtrated and washed with CHCl_3_. Then H_2_O was added and organic layer was extracted and dried under reduced pressure.

1. **Investigation of residual Mn in GO**

20.0 mg of GO was dispersed into the mixture of 9.0 mL of 60% aq. HNO_3_ and 1.0 mL of 30% aq. H_2_O_2_, and stirred for 60 min. After the treatment, the reaction mixture was filtrated and the resulting solution was diluted to appropriate concentration, then atomic absorption of Mn was measured using stock solution of Mn (0, 1.0, 2.0, 3.0, 5.0, 7.5, 10 μM) for calibration.

Table S3. Investigation of the amount of residual Mn in GO


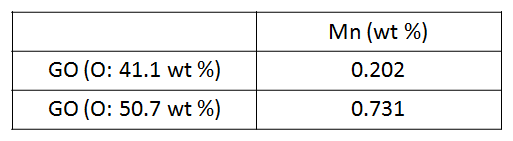


1. **Evaluation of oxidation activity of GO which has several oxygen content**
   1. **Oxidation of benzylalcohol**


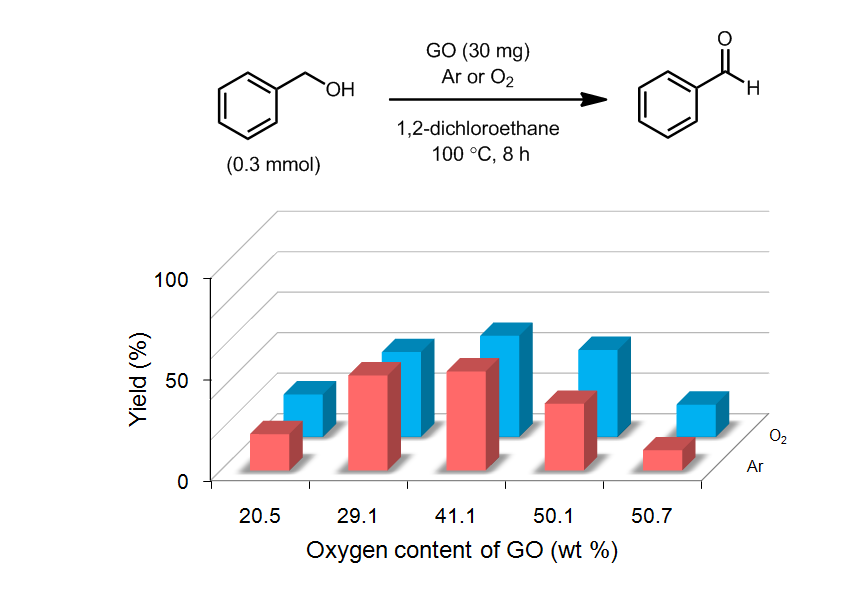
To the solution of 1,2-dichloroethane (0.2 mL), GO (30.0 mg) and benzylalcohol (32.4 mg, 0.3 mmol) were added under Ar or O_2_ atmosphere and stirred at 100 °C for 8 h. After the reaction, the reaction mixture was analyzed by GC using *n*-dodecane as an internal standard.

Figure S1. Oxidation of benzylalcohol to benzaldehyde using GO.

**9.2 Oxidative homocoupling of benzylamine**

To the solution of 1,2-dichloroethane (0.2 mL), GO (10.0 mg) and benzylamine (32.1 mg, 0.3 mmol) were added under Ar or O_2_ atmosphere and stirred at 100 °C for 8 h. After the reaction, the reaction mixture was analyzed by GC using *n*-dodecane as an internal standard.


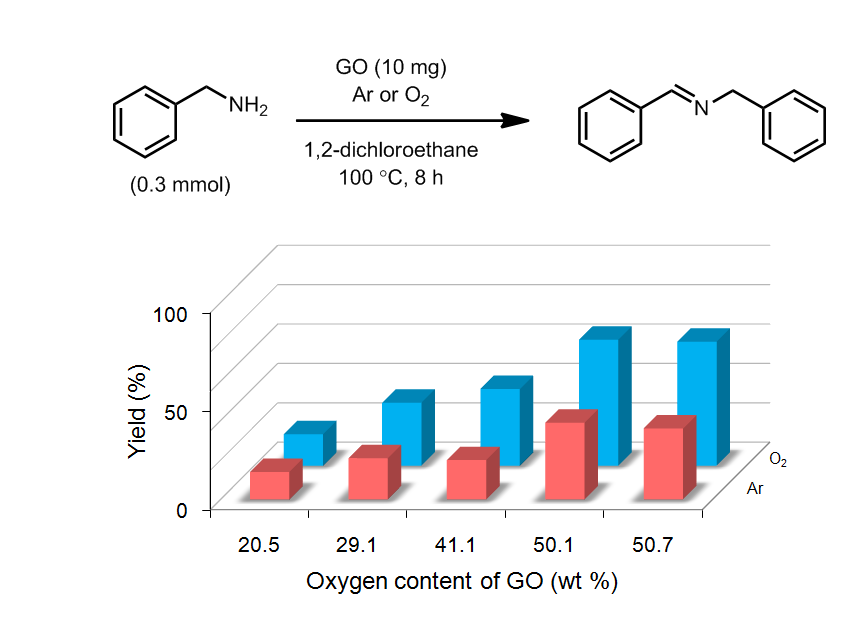


Figure S2. Oxidative homocoupling of benzylamine using GO.

**9.3 C-H oxidation of diphenylmethane**


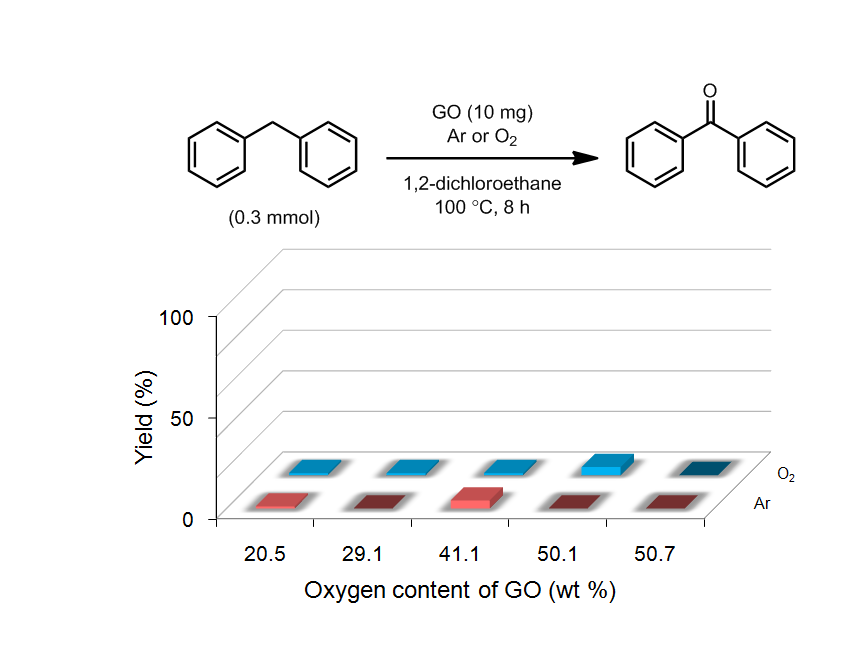
To the solution of 1,2-dichloroethane (0.2 mL), GO (10.0 mg) and diphenylmethane (50.5 mg, 0.3 mmol) were added under Ar or O_2_ atmosphere and stirred at 100 °C for 8 h. After the reaction, the reaction mixture was analyzed by GC using *n*-dodecane as an internal standard.

Figure S3. C-H oxidation of diphenylmethane using GO.

**9.4 Oxidative dehydrogenative coupling reaction using GO**


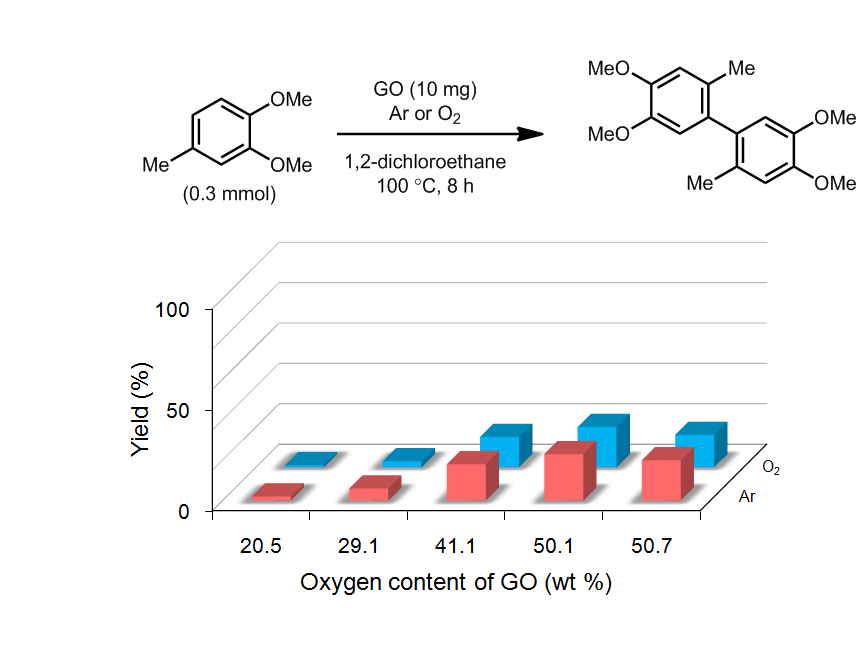
To the solution of 1,2-dichloroethane (0.2 mL), GO (10.0 mg) and 3,4-dimethoxytoluene (45.7 mg, 0.3 mmol) were added under Ar or O_2_ atmosphere and stirred at 100 °C for 8 h. After the reaction, the reaction mixture was analyzed by GC using *n*-dodecane as an internal standard.

Figure S4. Oxidative dehydrogenative coupling reaction using GO.

**10. Optimization of reaction condition**

**General procedure for Table S5-8.**

To the solution of 1,2-dichloroethane (0.2 mL), GO (10.0 mg), 3,4-dimethoxytoluene (45.7 mg, 0.3 mmol) and BF_3_·OEt_2_ (42.6 mg, 0.3 mmol) were added under Ar atmosphere and stirred at 60 °C for 8 h. After the reaction, the reaction mixture was analyzed by GC using *n*-decane as an internal standard.


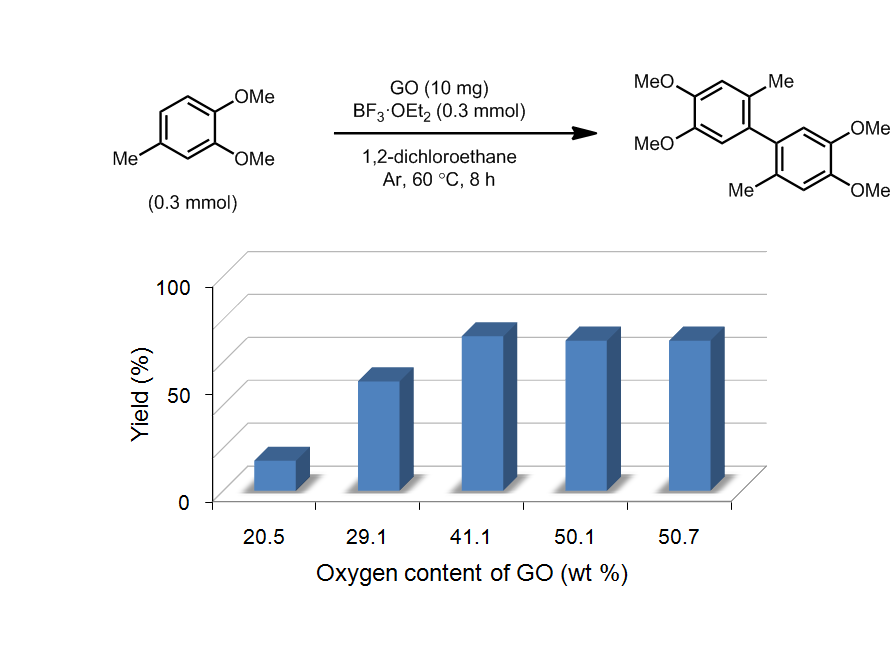


Figure S5. The screening of the oxidative coupling reactions with different oxygen content GO.


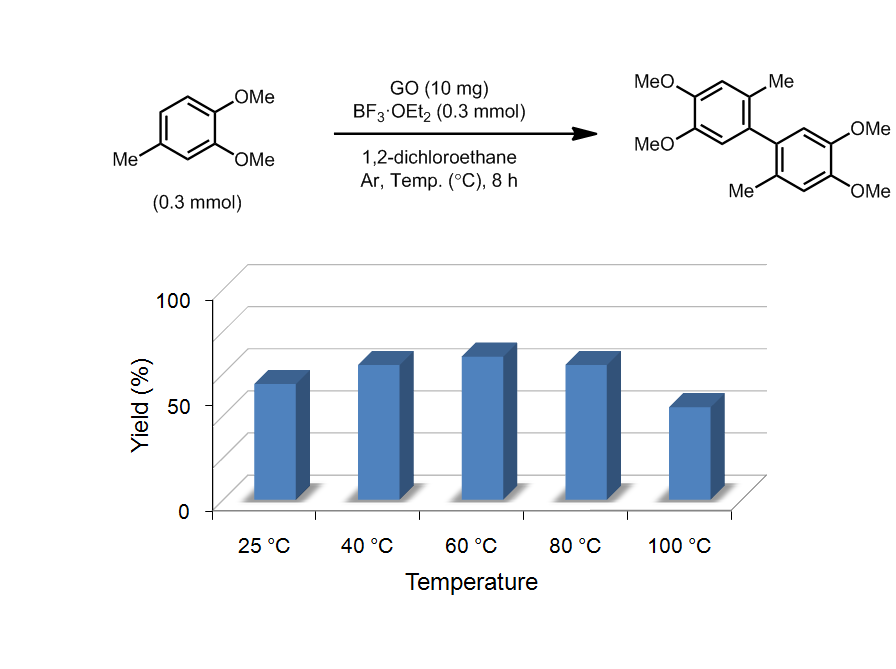


Figure S6. The screening of reaction temperature.


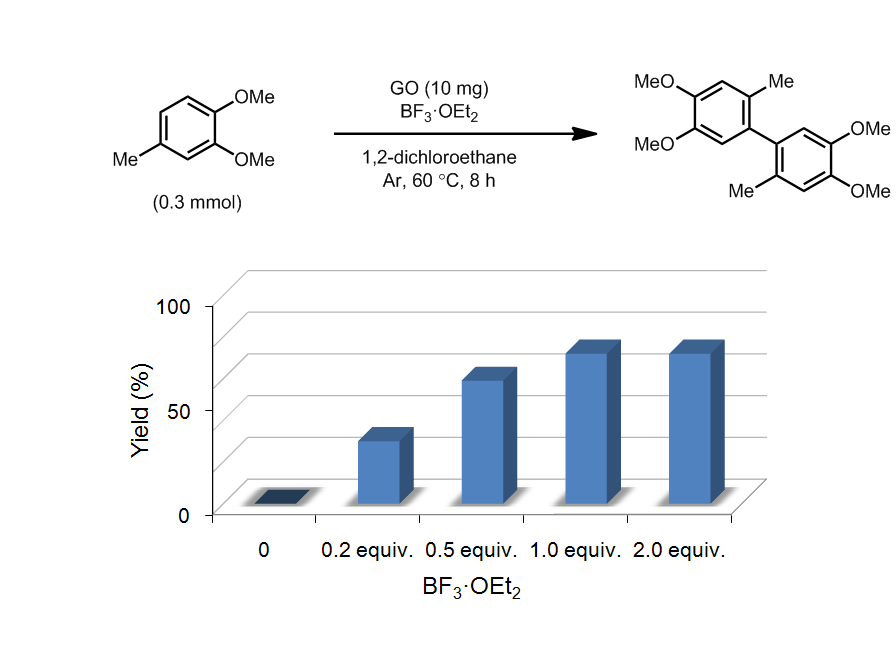


Figure S7. The screening of the amount of BF_3_·OEt_2_.


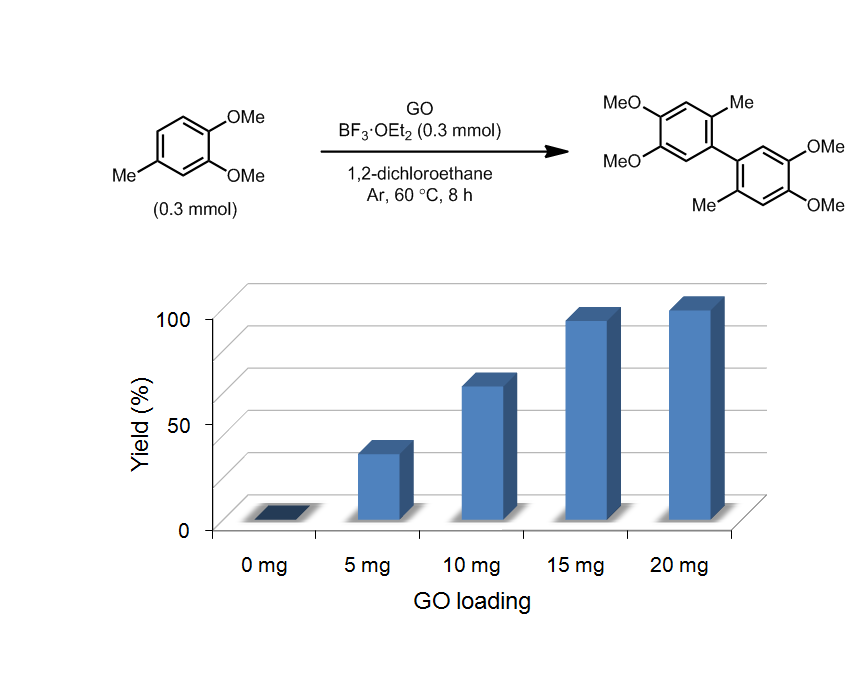


Figure S8. The screening of the loading amount of GO.

1. **The effect of water addition**


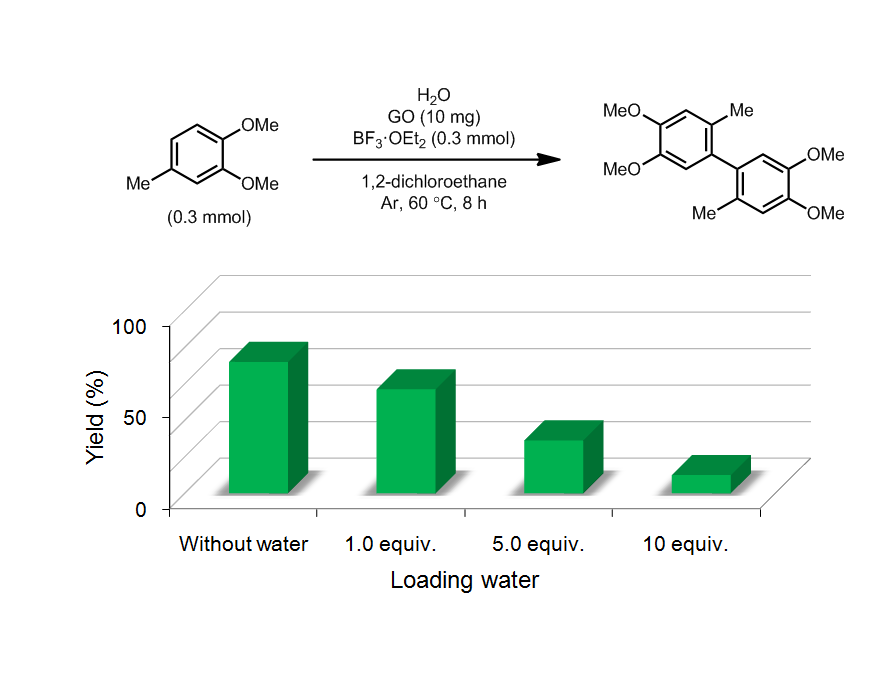
To the solution of 1,2-dichloroethane (0.2 mL), GO (10.0 mg), 3,4-dimethoxytoluene (45.7 mg, 0.3 mmol), BF_3_·OEt_2_ (42.6 mg, 0.3 mmol) and water were added under Ar atmosphere and stirred at 60 °C for 8 h. After the reaction, the reaction mixture was analyzed by GC using *n*-decane as an internal standard.

Figure S9. The Investigation of the effect of water addition.

1. **The effect other oxidant**

To the solution of 1,2-dichloroethane (0.5 mL), GO (10.0 mg), 3,4-dimethoxytoluene (152 mg, 1.0 mmol), BF_3_·OEt_2_ (142 mg, 1.0 mmol) and oxidant (1.0 mmol) were added and stirred at 60 °C for 8 h. After the reaction, the reaction mixture was analyzed by GC using *n*-decane as an internal standard.

Table S4. Trial for catalytic reaction using other oxidant.

1. **Determination water generation by Karl-Fischer method**

To the solution of 1,2-dichloroethane (4.0 mL), GO (200 mg), 3,4-dimethoxytoluene (913 mg, 6.0 mmol) and BF_3_·OEt_2_ (852 mg, 6.0 mmol) were added under Ar atmosphere and stirred at 60 °C for 8 h. After the reaction, the reaction mixture was filtrated and Karl-Fisher analysis was performed (sample 1). The control experiment was also performed without 3,4-dimethoxytoluene (sample 2). As the result of analysis, 1.88 mmol of water was produced during the reaction.

Table S5. Water generation in the course of the reaction.


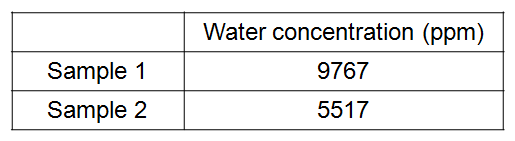


1. **Comparison of GO and ^13^C NMR spectra of several compounds which has epoxy and hydroxyl group at 30-100 ppm.**


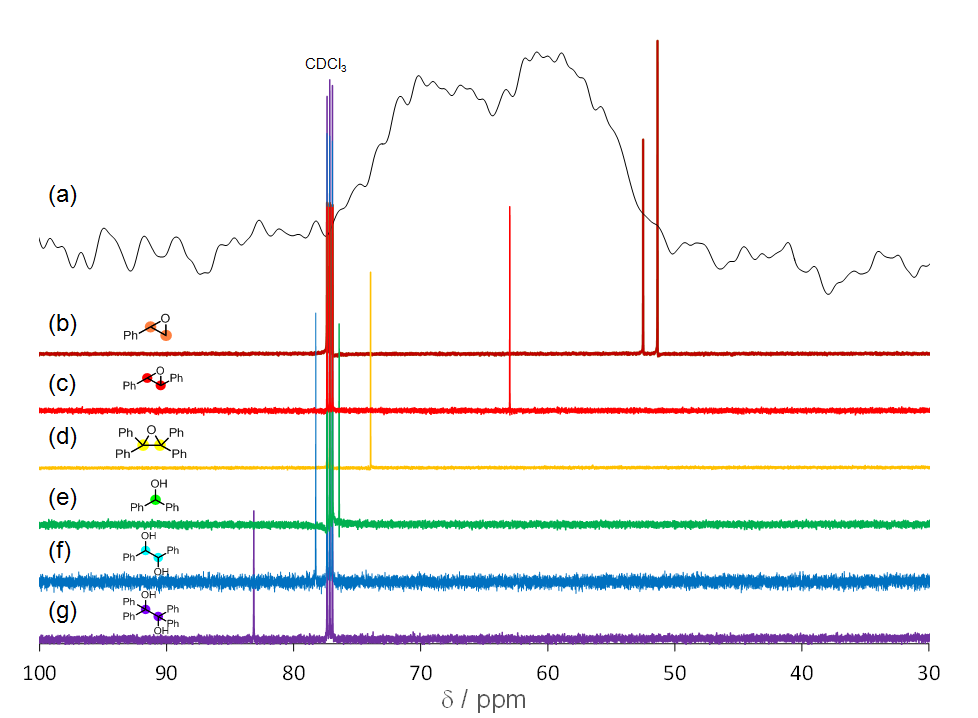


Figure S10. (a) ^13^C Solic state NMR spectrum of GO (O: 41.1 w%), ^13^C NMR spectra of (b) stylene oxide, (c) trans-stilbene oxide, (d) 2,2,3,3-tetraphenyl-oxirane, (e) benzhydrol, (f) 1,2-diphenyl-1,2-ethanediol and (g) 1,1,2,2-tetraphenyl-1,2-ethanediol in CDCl_3_.

^13^C NMR spectra of GO was compared with that of several model compounds. Epoxy group were detected at 50-75 ppm, and hydroxyl group were shown over 75ppm when model compounds were measured. However some scientists regards the GO peak at 60 and 70ppm as epoxy and hydroxyl group, this experimental data shows that the peak at 70ppm can be assigned as tetra-substituted epoxide.

1. **The reaction with radical scavenger (1)**

To the solution of 1,2-dichloroethane (0.2 mL), GO (10.0 mg), 3,4-dimethoxytoluene (45.7 mg, 0.3 mmol), 2,2,6,6-tetramethylpiperodine 1-oxyl (46.9 mg, 0.3 mmol) and BF_3_·OEt_2_ (42.6 mg, 0.3 mmol) were added under Ar atmosphere and stirred at 60 °C for 8 h. After the reaction, the reaction mixture was analyzed by GC using *n*-decane as an internal standard.

1. **The interaction between BF_3_·OEt_2_ and GO (FT-IR)**

**16.1 Preparation of the mixture of 3,4-dimthoxytoluene and BF_3_·OEt_2_ (Figure S11, (a))**

To the solution of 3,4-dimethoxytoluene (761 mg, 5.0 mmol) and BF_3_·OEt_2_ (710 mg, 5.0 mmol) were added under Ar atmosphere and stirred at 60 °C for 1 h.


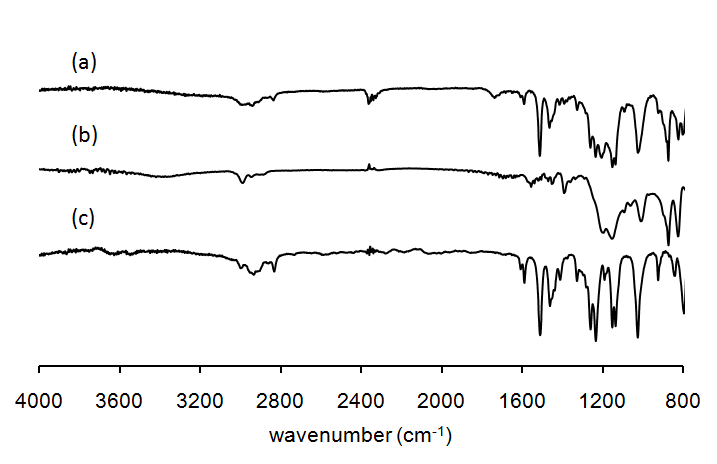


Figure S11. FT-IR spectra of (a) the mixture of 3,4-dimethoxytoluene and BF_3_·OEt_2_, (b) BF_3_·OEt_2_ and (c) 3,4-dimethoxytoluene.

**16.2** **Preparation of GO treated with BF_3_·OEt_2_ (Figure S12, (a))**

To the solution of BF_3_·OEt_2_ (1.0 mL) and GO (100 mg) were added under Ar atmosphere and stirred at 60 °C for 1 h. After the reaction, reaction mixture was filtrated and dry under reduced pressure.


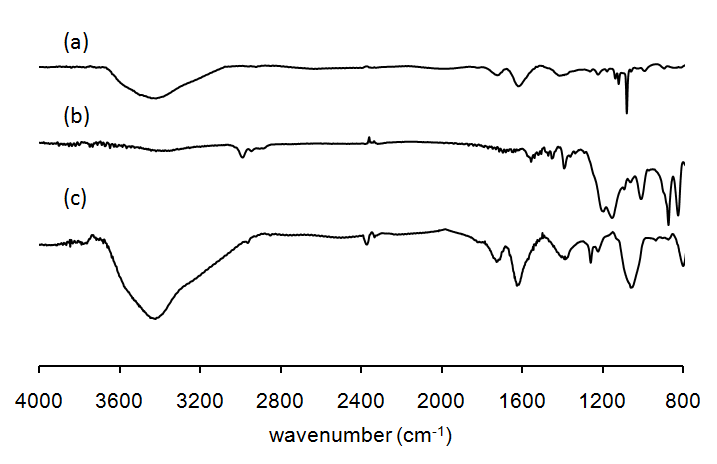


Figure S12. FT- IR spectra of (a) GO treated with BF_3_·OEt_2_, (b) BF_3_·OEt_2_ and (c) GO.

1. **The interaction between BF_3_·OEt_2_ and GO (^1^H NMR)**

**17.1 Preparation of the mixture of 3,4-dimethoxytoluene and BF_3_·OEt_2_ (Figure S13, (e))**

To the solution of CDCl_3_ (0.5 mL), 3,4-dimethoxytoluene (45.7 mg, 0.3 mmol) and BF_3_·OEt_2_ (42.6 mg, 0.3 mmol) were added under Ar atmosphere and stirred at 60 °C for 1 h.

**17.2 Preparation of the mixture of GO and BF_3_·OEt_2_ (Figure S13, (f))**

To the solution of CDCl_3_ (0.5 mL), GO (10.0 mg) and BF_3_·OEt_2_ (42.6 mg, 0.3 mmol) were added under Ar atmosphere and stirred at 60 °C for 1 h.

**17.3 Preparation of the mixture of GO and BF_3_·OEt_2_ (Figure S13, (g))**

To the solution of CDCl_3_ (0.5 mL), 3,4-dimethoxytoluene (45.7 mg, 0.3 mmol), GO (10.0 mg) and BF_3_·OEt_2_ (42.6 mg, 0.3 mmol) were added under Ar atmosphere and stirred at 60 °C for 1 h.


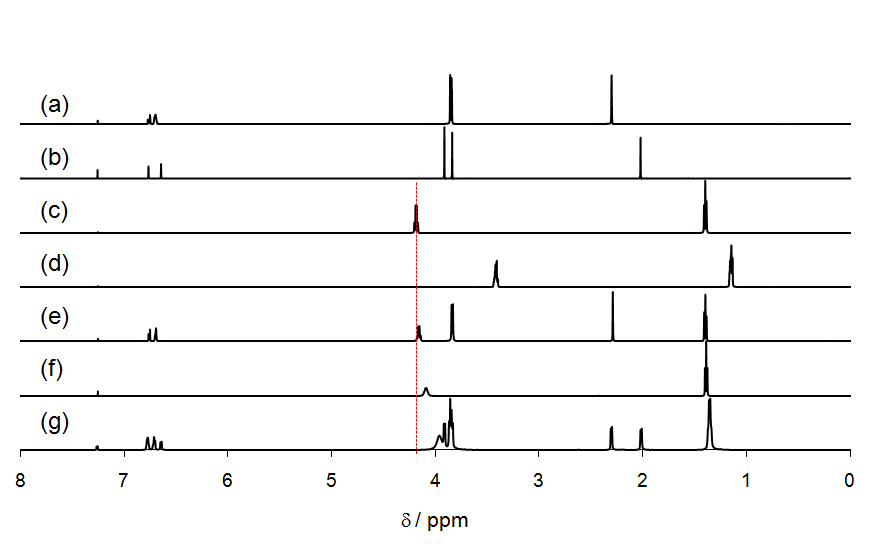


Figure S13. NMR spectra of (a) 3,4-dimethoxytoluene, (b) 4,​4',​5,​5'-​tetramethoxy-​2,​2'-​dimethyl-1,​1'-​biphenyl, (c) BF_3_·OEt_2_, (d) diethylether, the mixture of (e) 3,4-dimethoxytoluene and BF_3_·OEt_2_, (f) GO and BF_3_·OEt_2_ and (g) 3,4-dimethoxytoluene, GO and BF_3_·OEt_2_.

1. **Kinetic isotope effect (2) and (3)**

^1^H NMR chart of dimer product.

**
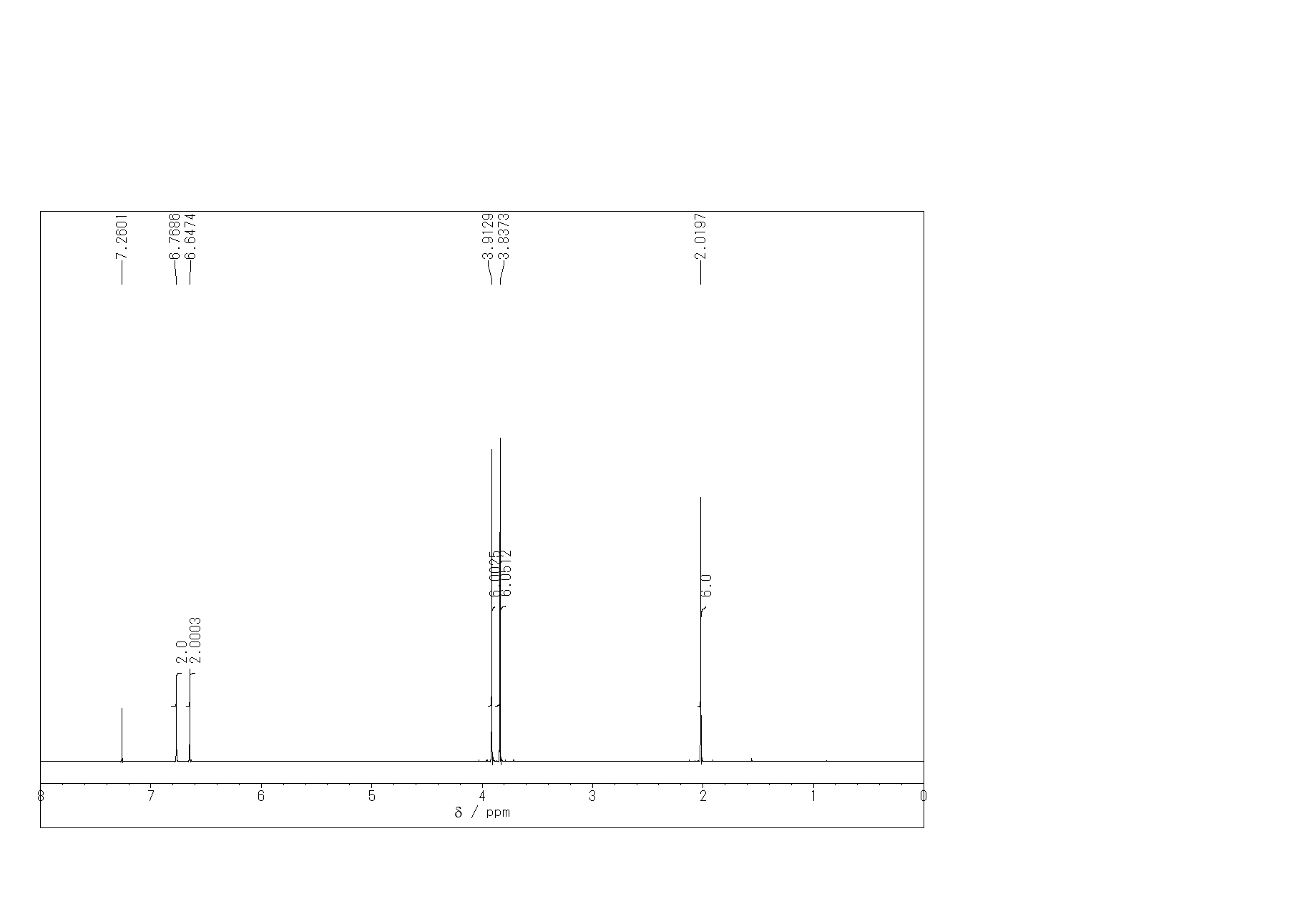
**

MS (EI) Calcd for C_18_H_22_O_4_ (EI) 302, Found 302

^1^H NMR chart of dimer products.


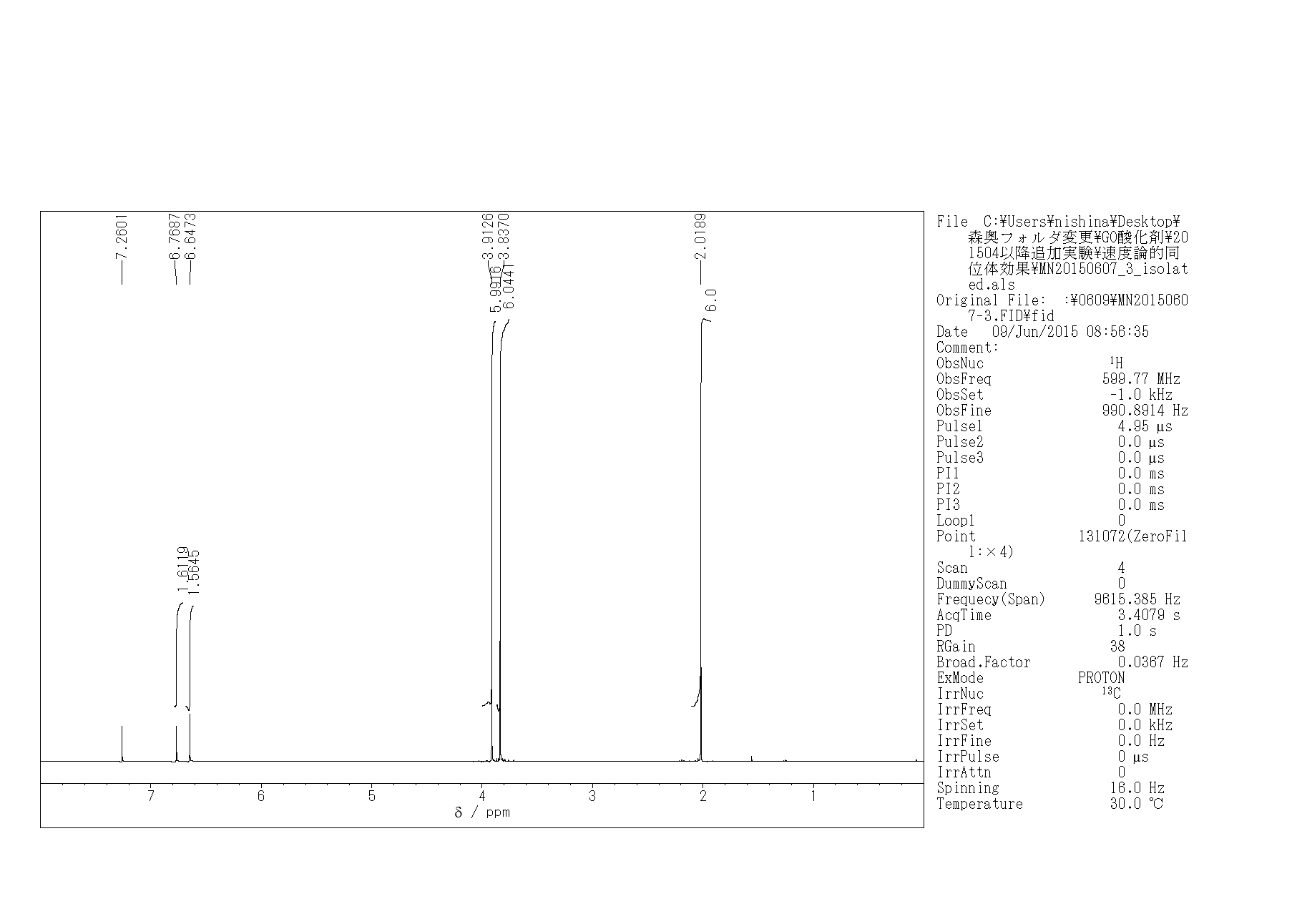


MS (EI), Found 303

1. **Comparison with other oxidant**

**19. 1 Comparison with hypervalent iodine compound (4)**

The reaction condition was refered to known method.^5^

The solution of BF_3_·OEt_2_ (42.6 mg, 0.3 mmol) in dichloromethane (5.0 mL) was added slowly to the solution of dichloromethane (5.0 mL), PhI(OAc)_2_ (55.3 mg, 0.17 mmol), and 6-deuterio-3,4-dimethoxytoluene (46.0 mg, 0.3 mmol) under Ar atmosphere at -40 °C and reaction mixture was stirred for 1.5 h. After the reaction, reaction mixture was quenched by AcOEt and water. The organic phase was concentrated under reduced pressure and purified by column chromatography (29.3 mg, 65% yield). Structures of product were determined by ^1^H NMR and EI-MS.

^1^H NMR chart of dimer product.


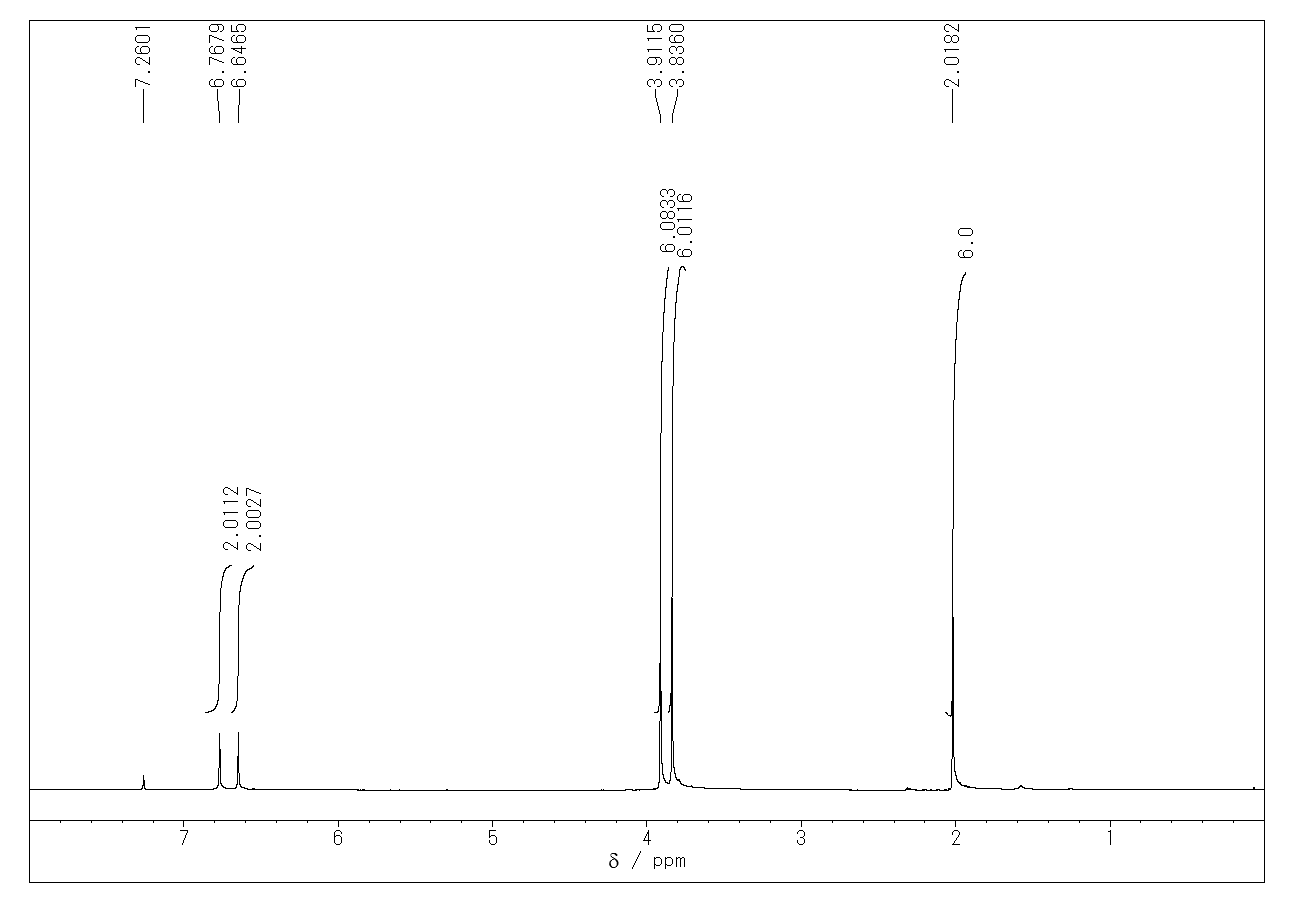


MS (EI) Calcd for C_18_H_22_O_4_ (EI) 302, Found 302

**19.2 Comparison with 2,3-dichloro-5,6-dicyano-p-benzoquinone (DDQ) (5)**

The reaction condition was refered to known method.^6^

To the solution of dichloromethane (0.5 mL), DDQ (68.1 mg), 6-deuterio-3,4-dimethoxytoluene (46.0 mg, 0.3 mmol) and BF_3_·OEt_2_ (425.7 mg, 3.0 mmol) were added under Ar atmosphere and stirred at rt for 10 min. After the reaction, reaction mixture was quenched by AcOEt and water. The organic phase was concentrated under reduced pressure and purified by column chromatography (32.0 mg, 70% yield). Structures of product were determined by ^1^H NMR and EI-MS.

^1^H NMR chart of dimer product.


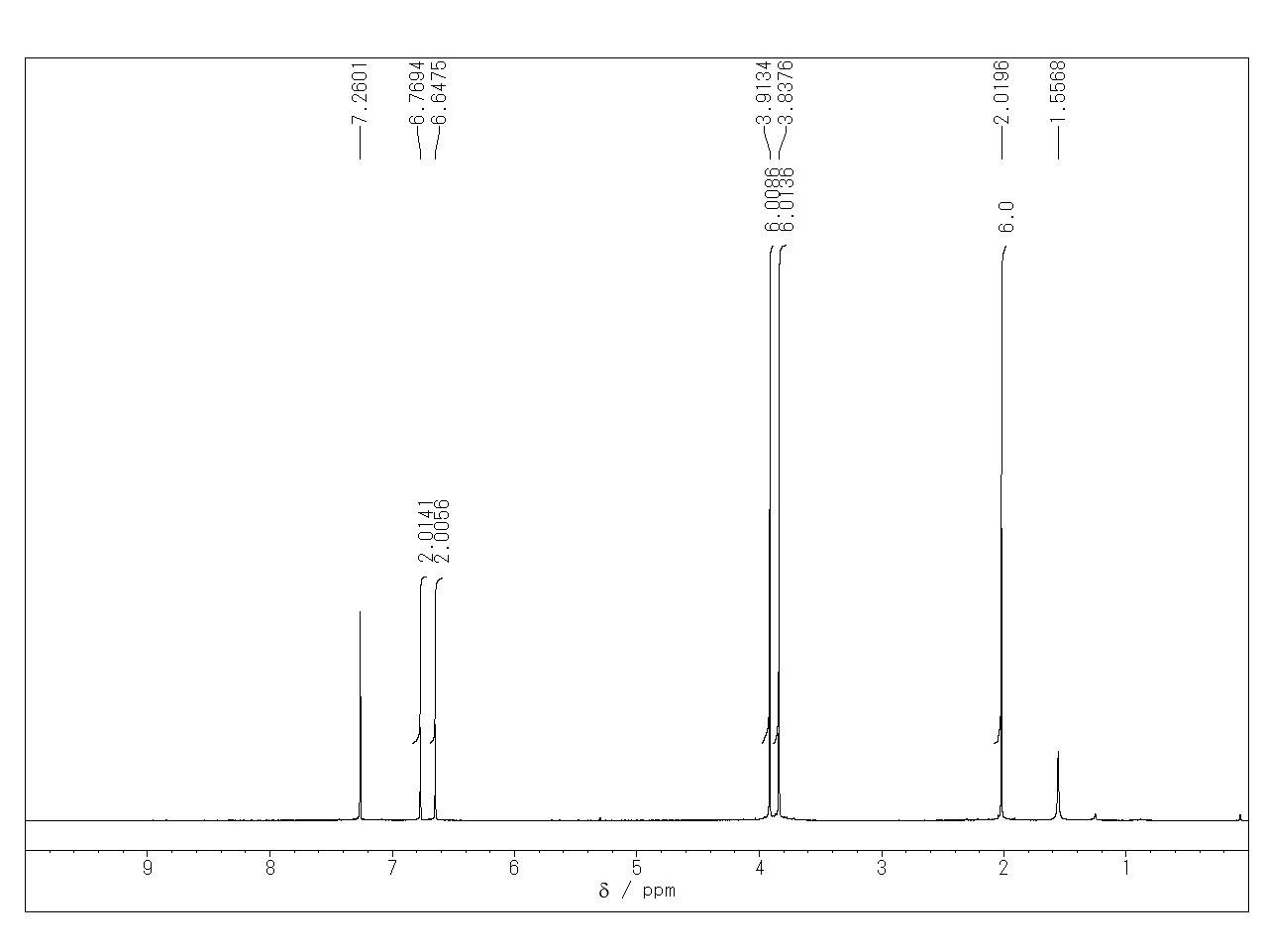


MS (EI) Calcd for C_18_H_22_O_4_ (EI) 302, Found 302

1. **H/D exchange experiment with D_2_O**

**20.1 H/D exchange experiment using GO (6)**

To the solution of 1,2-dichloroethane (0.2 mL), GO (10.0 mg), BF_3_·OEt_2_ (42.6 mg, 0.3 mmol), 3,4-dimethoxytoluene (45.7 mg, 0.3 mmol) and D_2_O (30.0 mg, 1.5 mmol) were added under Ar atmosphere and stirred at 60 °C for 12 h. After the reaction, reaction mixture was quenched by AcOEt and water. The organic phase was concentrated under reduced pressure and purified by column chromatography. Structures of product were determined by ^1^H NMR and EI-MS.

^1^H NMR chart of 6-deuterio-3,4-dimethoxytoluene.

**
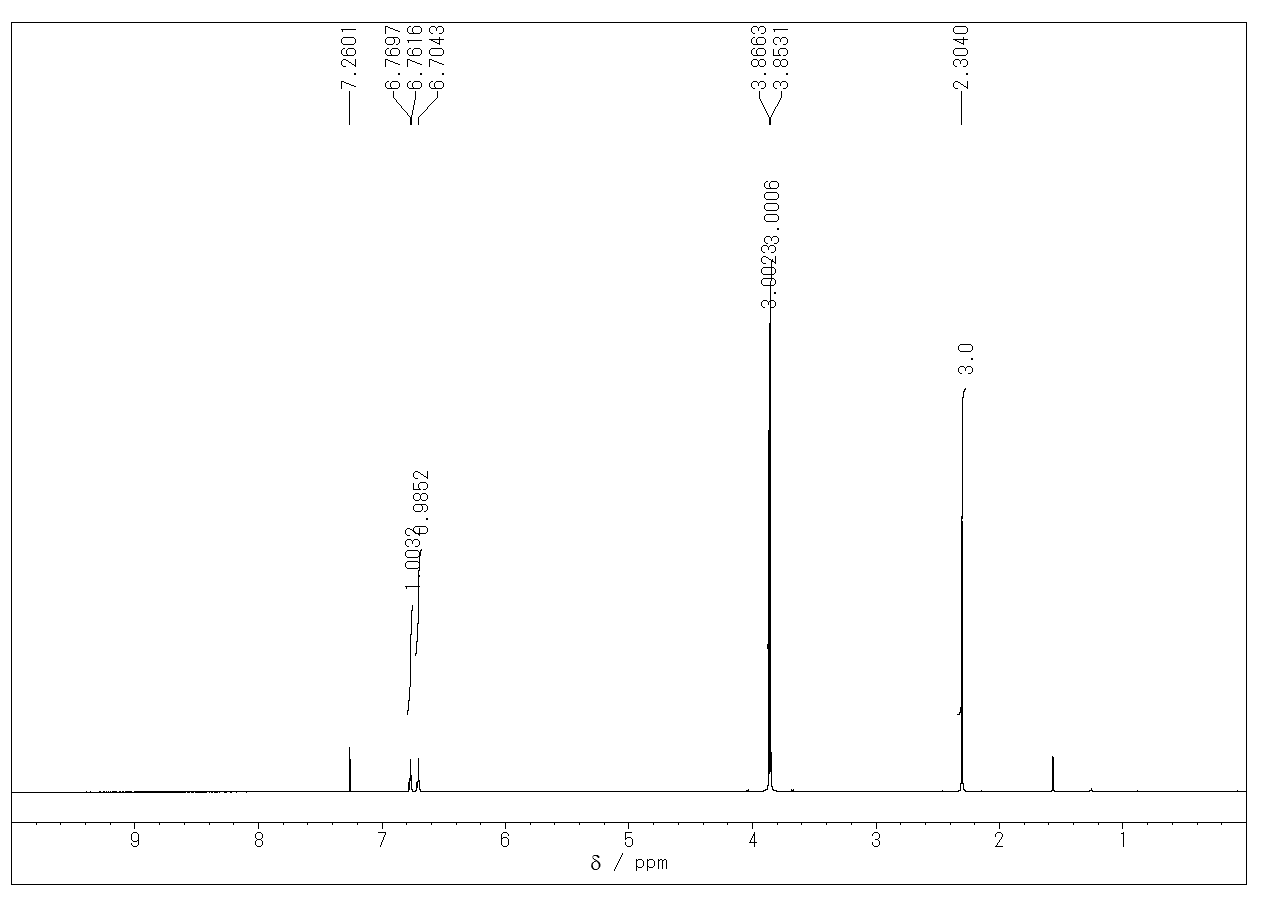
**

^1^H NMR chart of dimer products.

**
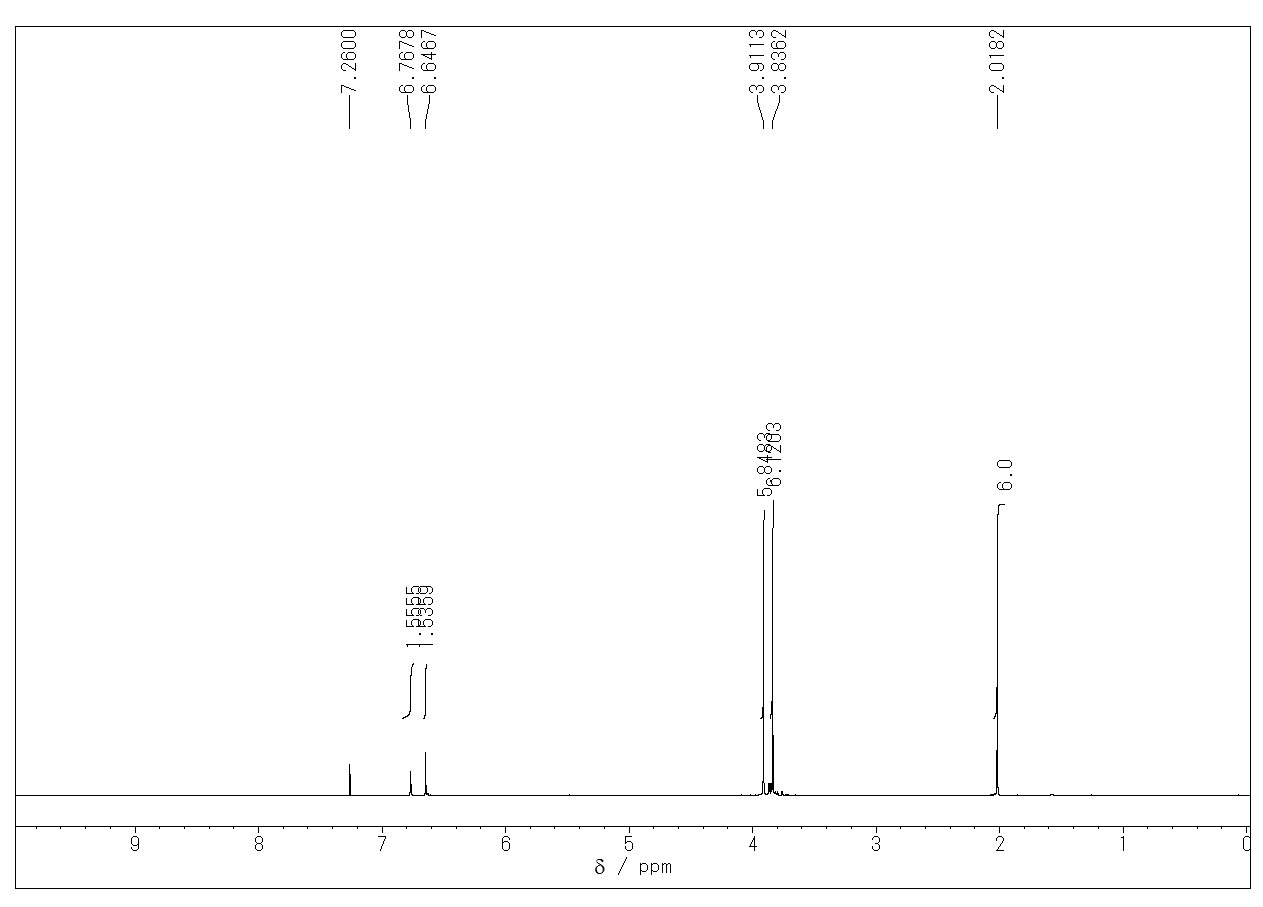
**

**20.2 H/D exchange experiment using GO (7)**

The solution of BF_3_·OEt_2_ (42.6 mg, 0.3 mmol) in dichloromethane (5.0 mL) was added slowly to the solution of dichloromethane (5.0 mL), PhI(OAc)_2_ (55.3 mg, 0.17 mmol), D_2_O (30.0 mg, 1.5 mmol) and 3,4-dimethoxytoluene (46.0 mg, 0.3 mmol) under Ar atmosphere at -40 °C and reaction mixture was stirred for 1.5 h. After the reaction, reaction mixture was quenched by AcOEt and water. The organic phase was concentrated under reduced pressure and purified by column chromatography. Structure of product were determined by ^1^H NMR and EI-MS. Deuterium labeled compound was not observed.

**21. Electrical conductivity and specific capacitance measurement**

**21.1 Procedure of surface area measurement.**

GO (10 mg) was added to 10-50 mL solution of known MB concentrations (13.1-130.7 mg/L). The mixture was sonicated (42 kHz, 130 W) for 90 min, then filtrated. The resulting solution was diluted to appropriate concentration and UV-Vis measurement was made at 664 nm using stock aqueous solution of MB (0, 1.1, 2.2, 3.4 mg/L) for calibration. The surface area was estimated considering 1.0 mg of adsorbed MB equates to a surface area of 2.54 m^2^.^7^ The surface area of GO (oxygen content: 50.7 wt %) was 894 m^2^/g.

**21.2 Procedure of electrical conductivity measurement.**

Each sample was pelletized before the measurement using four-point probe. To investigate the electrical conductivity of GO, the average resistance was measured at 3 sampling points. The specific resistance was calculated according to

ρ = *RFL*

,where ρ is the specific resistance (Ωcm), *R* is the pellet thickness (cm), *F* is the correction coefficient which was determined from the distance between the probes attached with the instruments and *L* is the measured pellet resistance (Ω). The electrical conductivity (Scm^-1^) was calculated reciprocal number of specific resistance.

**21.3 Procedure of specific capacitance measurement.**

Samples (15.0 mg) was sandwiched with Ni meshes and dried at 120 °C for 6 h. Each sample was directly used as electrodes in a three-electrode test cell using 1.0 M KOH aqueous solution as an electrolyte. The gravimetric capacitance of the electrodes was calculated according to

*C =* ʃ*idV*/*mvΔV*

,where *C* is the specific capacitance (Fg^-1^), *i* is current (A), *V* is the potential (V), *v* is the scan rate (Vs^-1^), and *m* is the mass of the active material (g). Calculation for ʃ*idV* was made using cyclic voltammograms of the second cycle.

Table S6. Conductivity and specific capacitance measurement of GO, recovered GO and hydrazine reduced GO.


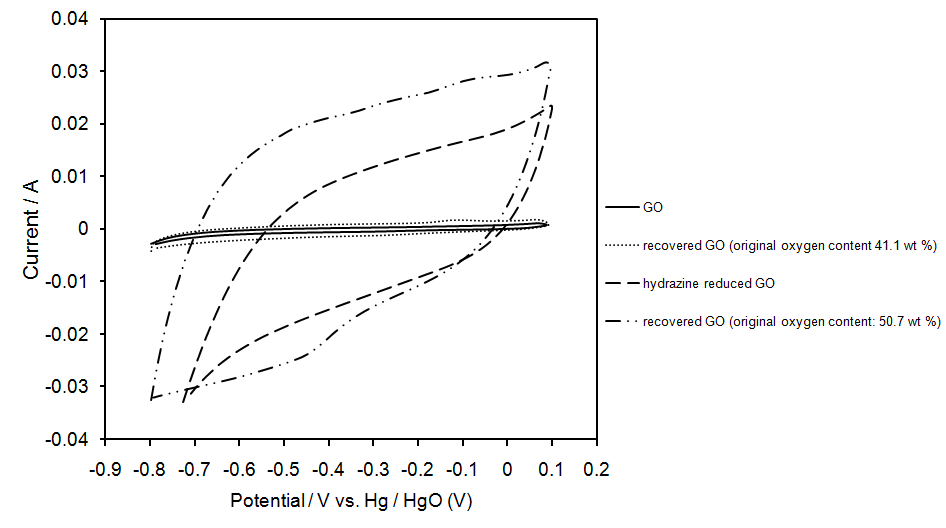


Figure S14. Cyclic voltammograms of GO in 1.0 M KOH.

**22. Product data**

4,4',5,5'-tetramethoxy-2,2'-dimethyl-1,1'-biphenyl (**2a**)

^1^H NMR (600 MHz, CDCl_3_) δ 6.77 (s, 2H), 6.65 (s, 2H), 3.91 (s, 6H), 3.84 (s, 6H), 2.02 (s, 6H)

2,2'-difluoro-4,4',5,5'-tetramethoxy-1,1'-biphenyl (**2b**)

^1^H NMR (600 MHz, CDCl_3_) δ 6.85 (s, 2H), 6.73 (s, 2H), 3.91 (s, 6H), 3.89 (s, 6H)

^13^C NMR (150 MHz, CDCl3) δ 154.13 (dd, *J* = 240.9, 1.4 Hz), 149.63 (d, *J* = 4.0 Hz), 149.59 (d, ­*J* = 4.3 Hz), 145.25-145.26 (m), 114.03 (dd, *J* = 12.6, 2.6 Hz), 113.60 (dd, *J*= 3.5, 3.2 Hz), 100.47 (t, *J* = 7.0 Hz), 100.28 (t, *J* = 7.0 Hz), 56.66 (s), 56.34 (s)

IR 2924, 2837, 1615, 1600, 1502, 1457, 1431, 1375, 1329, 1258, 1246, 1209, 1190, 1171, 1148, 1028, 968, 953, 825, 776, 716, 690, 633 cm^-1^

HRMS (FAB+) Calcd for C_16_H_17_F_2_O_4_ (FAB+) 311.1095, Found 311.1116

2,2'-dichloro-4,4',5,5'-tetramethoxy-1,1'-biphenyl (**2c**)

^1^H NMR (600 MHz, CDCl_3_) δ 6.96 (s, 2H), 6.77 (s, 2H), 3.92 (s, 6H), 3.87 (s, 6H)

^13^C NMR (150 MHz, CDCl_3_) δ 56.32, 56.33, 112.38, 114.05, 125.02, 130.17, 147.59, 149.23

IR 2931, 2833, 1600, 1502, 1434, 1397, 1325, 1246, 1205, 1167, 1054, 1028, 968, 870, 784, 713 cm^-1^

HRMS (FAB+) Calcd for C_16_H_17_Cl_2_O_4_ (FAB+) 345.0474, Found 345.0448

2,2'-dibromo-4,4',5,5'-tetramethoxy-1,1'-biphenyl (**2d**)

^1^H NMR (600 MHz, CDCl_3_) δ 7.11 (s, 2H), 6.76 (s, 2H), 3.92 (s, 6H), 3.87 (s, 6H)

4,4',5,5'-tetrabuthoxy-2,2'-dimethyl-1,1'-biphenyl (**2g**)

^1^H NMR (600 MHz, CDCl_3_) δ 6.76 (s, 2H), 6.66 (s, 2H), 4.01-4.03 (m, 4H), 3.94-3.96 (m, 4H), 1.98 (s, 6 H), 1.76-1.83 (m, 8H), 1.47-1.53 (m, 8H), 0.94-1.01 (m, 12H)

^13^C NMR (150 MHz, CDCl_3_) δ 148.11, 146.71, 133.88, 128.61, 116.05, 115.52, 69.37, 69.16, 31.64, 31.58, 19.44, 19.38, 14.08, 14.05

IR 2958, 2924, 2871, 2845, 1604, 1502, 1465, 1431, 1401, 1378, 1329, 1246, 1209, 1156, 1111, 1062, 968, 953, 851, 825, 799 776, 727, 697, 633, 622 cm^-1^

HRMS (FAB+) Calcd for C_30_H_47_O_4_ (FAB+) 471.3474, Found 471.3596

2,2',4,4',6,6'-hexamethoxy-1,1'-biphenyl (**4a**)

^1^H NMR (600 MHz, CDCl_3_) δ 6.25 (s, 4H), 3.85 (s, 6H), 3.72 (s, 12Hz)

2,3,6,7,10,11-hexamethoxytriphenylene (**4b**)

^1^H NMR (600 MHz, CDCl_3_) δ 7.82 (s, 6H),  4.13 (s, 18H)

2,2'-dimethoxy-1,1'-binaphthalene (**4e**)

^1^H NMR (600 MHz, CDCl_3_) δ 7.98 (d, 2H, *J* = 9.0 Hz), 7.86 (d, 2H, *J* = 8.2 Hz) , 7.46 (d, 2H, *J* = 9.1 Hz) , 7.30-7.33 (m, 2H) , 7.19-7.22 (m, 2H) , 7.10 (d, 2H, *J*= 8.5 Hz)  3.77 (s, 6H)

[1,1'-binaphthalene]-2,2'-diol (**4f**)

^1^H NMR (600 MHz, CDCl_3_) δ 7.99 (d, 2H, *J* = 9.0 Hz), 7.90 (d, 2H, *J* = 8.0 Hz), 7.38-7.40 (m, 4H), 7.26-7.37 (m, 2H), 7.16 (d, 2H, *J* = 8.4 Hz), 5.03 (s, 2H)

2,3,6,7-tetramethoxy triphenylene (**4g**)

^1^H NMR (600 MHz, CDCl_3_) δ 8.49−8.50 (m, 2H), 8.00 (s, 2H), 7.78 (s, 2H), 7.60-7.61 (m, 2H), 4.13 (s, 6H), 4.12 (s, 6H)

2,3,6,7-tetramethoxy phenanthrene (**4h**)

^1^H NMR (600 MHz, CDCl_3_) δ 7.81 (s, 2H), 7.56 (s, 2H), 7.23 (s, 2H), 4.13 (s, 6H), 4.04 (s, 6H)

1-(2,4,6-trimethoxyphenyl)naphthalene (**4i**)

^1^H NMR (600 MHz, CDCl_3_) δ 7.86 (d, 1H, *J* = 8.9 Hz), 7.80-7.81 (m, 1H), 7.36-7.39 (m, 2H), 7.30-7.31 (m, 2H), 6.32 (s, 2H), 3.91 (s, 3H), 3.85 (s, 3H), 3.64 (s, 6H)

^13^C NMR (150 MHz, CDCl_3_) δ 55.47, 56.10, 57.30, 76.95, 77.16, 77.37, 91.33, 106.53, 114.70, 118.51, 123.38, 125.40, 125.99, 127.99, 129.00, 129.33, 134.18, 155.11, 159.31, 161.17

IR 2947, 2842, 1585, 1509, 1457, 1410, 1377, 1329, 1267, 1254, 1223, 1201, 1151, 1128, 1060, 1035, 1020, 971, 944, 903, 807 cm^-1^

HRMS (FAB+) Calcd for C_20_H_21_O_4_ (FAB+) 325.1440, Found 325.1317

2-methoxy-1-(4,5-dimethoxy-2-methylphenyl)naphthalene (**4J)**

^1^H NMR (600 MHz, CDCl_3_) δ 7.90 (d, 1H, *J* = 9.0 Hz), 7.83-7.85 (m, 1H), 7.39 (d, 1H, *J* = 9.0 Hz), 7.34-7.36 (m, 3H), 6.89 (s, 1H), 6.73 (s, 1H), 3.97 (s, 3H), 3.88 (s, 3H), 3.82 (s, 3H), 1.96 (s, 3H)

^13^C NMR (150 MHz, CDCl_3_) δ 19.36, 55.88, 56.03, 56.69, 76.92, 77.13, 77.34, 113.02, 113.71, 114.10, 123.56, 124.39, 125.25, 126.45, 127.78, 127.93, 129.03, 129.09, 129.75, 133.84, 146.83, 148.09, 154.00

IR 3000, 2964, 2939, 2848, 1501, 1451, 1335, 1254, 1204, 1145, 1093, 1064, 1009, 903, 857, 838, 812, 741, 696, 652, 555 cm^-1^

HRMS (FAB+) Calcd for C_20_H_20_O_3_ (FAB+) 309.1491, Found 309.1458

**23. Synthesis of substrate**

**4-chloro-1,2-dimethoxybenzene (1c)**

To a solution of 4-chloro-2-methoxyphenol (0.61 mL, 5.0 mmol) and K_2_CO_3_ (1.4 g, 10 mmol) in 1,4-dioxane was added CH_3_I (0.60 mL, 10 mmol). The solution was refluxed overnight. The reaction was work-up by water and organic phase was extracted with AcOEt (20 mL) and water (20 mL). The organic layer was washed with brine (20 mL), dried over MgSO_4_ and concentrated under reduced pressure. The residue was purified by column chromatography to afford 4-chloro-1,2-dimethoxybenzene as a pale yellow oil (811.2 mg, 94%)

^1^H NMR (600 MHz, CDCl_3_) δ 6.85 (dd, 1H, *J* = 8.5, 2.4 Hz), 6.82 (d, 1H, *J* = 2.4 Hz), 6.74 (d, 1H, *J* = 8.5 Hz), 3.83 (s, 1H), 3.82 (s, 1H)

^13^C NMR (150 MHz, CDCl_3_) δ 56.04, 56.07, 76.95, 77.16, 77.37, 111.90, 112.01, 120.28, 125.61, 147.82, 149.56

**3,4-dibuthoxyltoluene (1g)**

To a solution of 4-methylcatechol (0.62 g, 5.0 mmol) and K_2_CO_3_ (4.15 g, 30 mmol) in 1,4-dioxane was added 1-bromobutane (3.2 mL, 30 mmol). The solution was refluxed overnight. The reaction was work-up by water and organic phase was extracted with AcOEt (20 mL) and water (20 mL). The organic layer was washed with brine (20 mL), dried over MgSO_4_ and concentrated under reduced pressure. The residue was purified by column chromatography to afford 3,4-dibuthoxyltoluene as a pale yellow oil (1.1 g, 92%)

^1^H NMR (600 MHz, CDCl_3_) δ 6.83−6.85 (m, 1H), 6.78 (d, 1H, *J* = 1.5 Hz), 6.73 (d, 1H, *J* = 8.0 Hz), 4.01-4.05 (m, 4H), 2.34 (s, 1H), 1.81-1.86 (m, 4H), 1.54-1.60 (m, 4H), 1.02-1.07 (m, 6H)

^13^C NMR (150 MHz, CDCl_3_) δ 13.66, 19.06, 20.70, 31.26, 31.29, 68.64, 69.08, 76.73, 76.95, 77.16, 114.26, 114.91, 120.89, 130.44, 146.80, 148.93

**6-deuterio-3,4-dimethoxytoluene (2aD)**

1.6 M of *n-*Buthyl lithium in *n-*hexane (10.4 mL) was added to the solution of 1-bromo-4,5-dimethoxy-2-methylbenzene (1.95 g, 8.5 mmol) in THF (20 mL) at -78 °C and stirred for 20 min. Then D_2_O (2.0 mL) was added to the reaction solution. The organic phase was extracted with AcOEt (50 mL) and water (30 mL). The organic layer was washed with brine (20 mL), dried over MgSO_4_ and concentrated under reduced pressure. The residue was purified by column chromatography to afford 6-deuterio-3,4-dimethoxytoluene with a pale yellow oil (1.1 g, 86 %)

^1^H NMR (600 MHz, CDCl_3_) δ 6.77 (s, 1H), 6.70 (s, 1H), 3.87 (s, 3H), 3.85 (s, 3H), 2.30 (s, 3H)

^13^C NMR (150 MHz, CDCl_3_) δ 21.1, 55.9, 56.1, 111.2, 112.6, 120.4, 120.6, 120.8, 130.4, 147.0, 148.8

IR 2995, 2943, 2833, 1604, 1461, 1389, 1321, 1254, 1216, 1156, 1024, 916, 870, 840, 746, 694 cm^-1^

HRMS (FAB+) Calcd for C_9_H_11ｓｓ_DO_2_ (FAB+) 153.0900, Found 153.0926


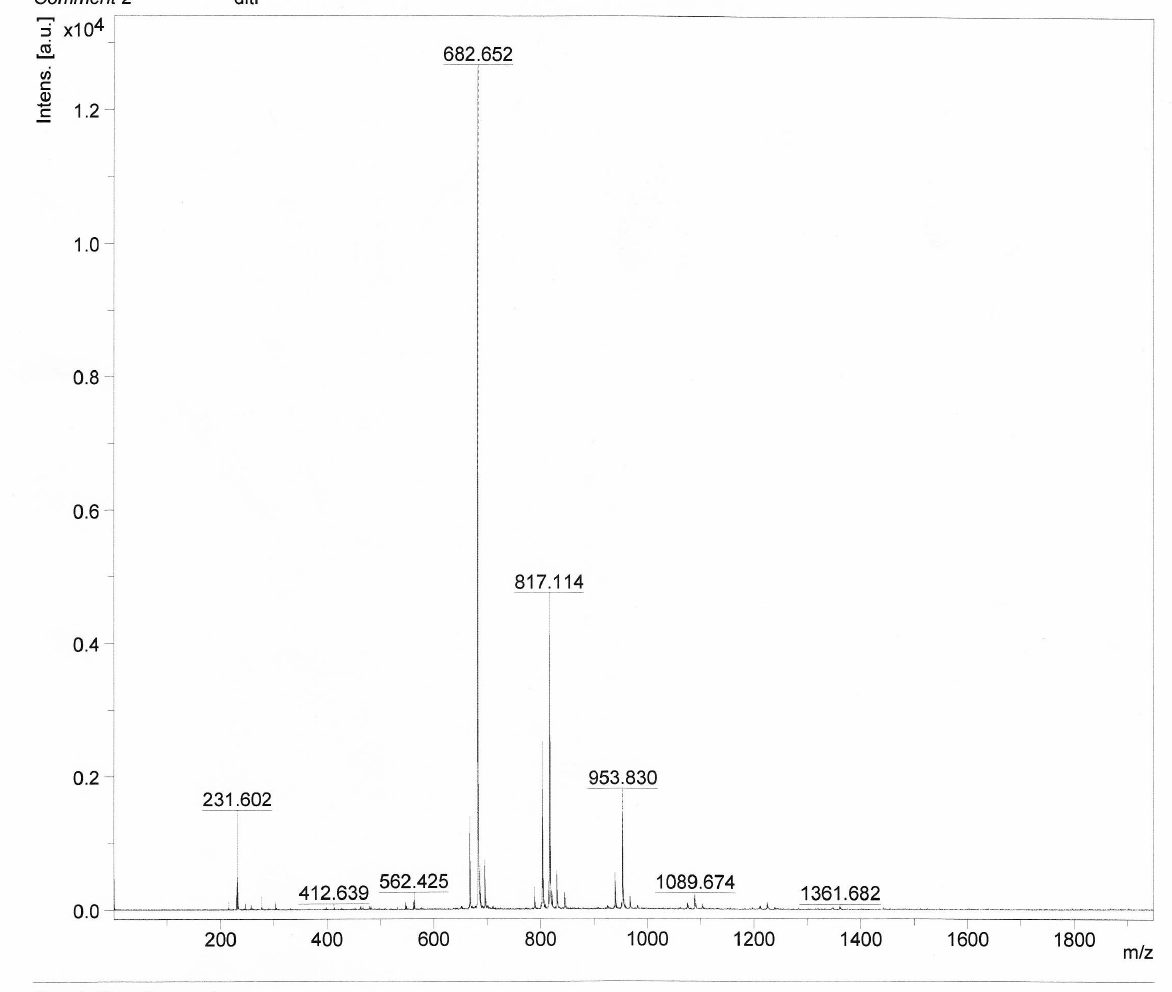
**25. Investigation of polymer compounds using MALDI-TOFMS**


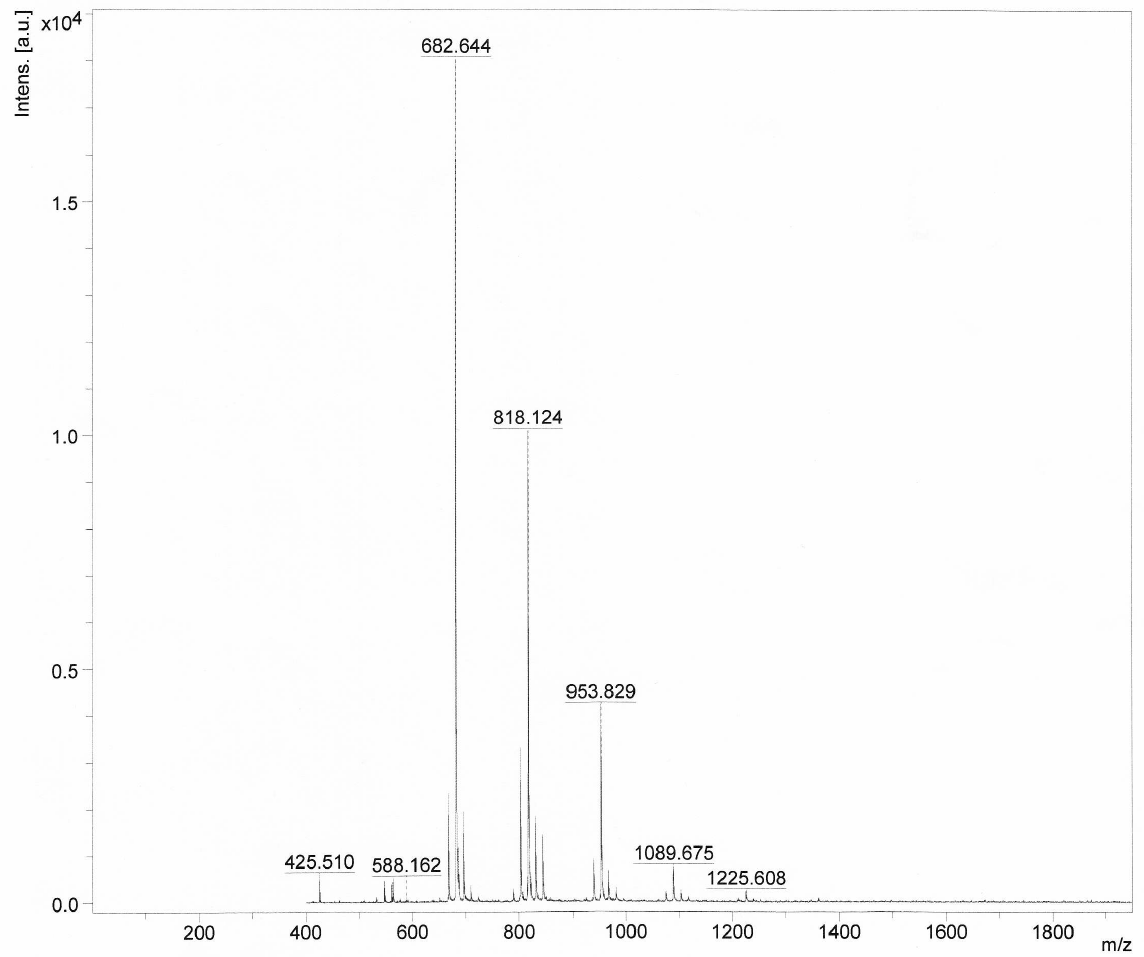


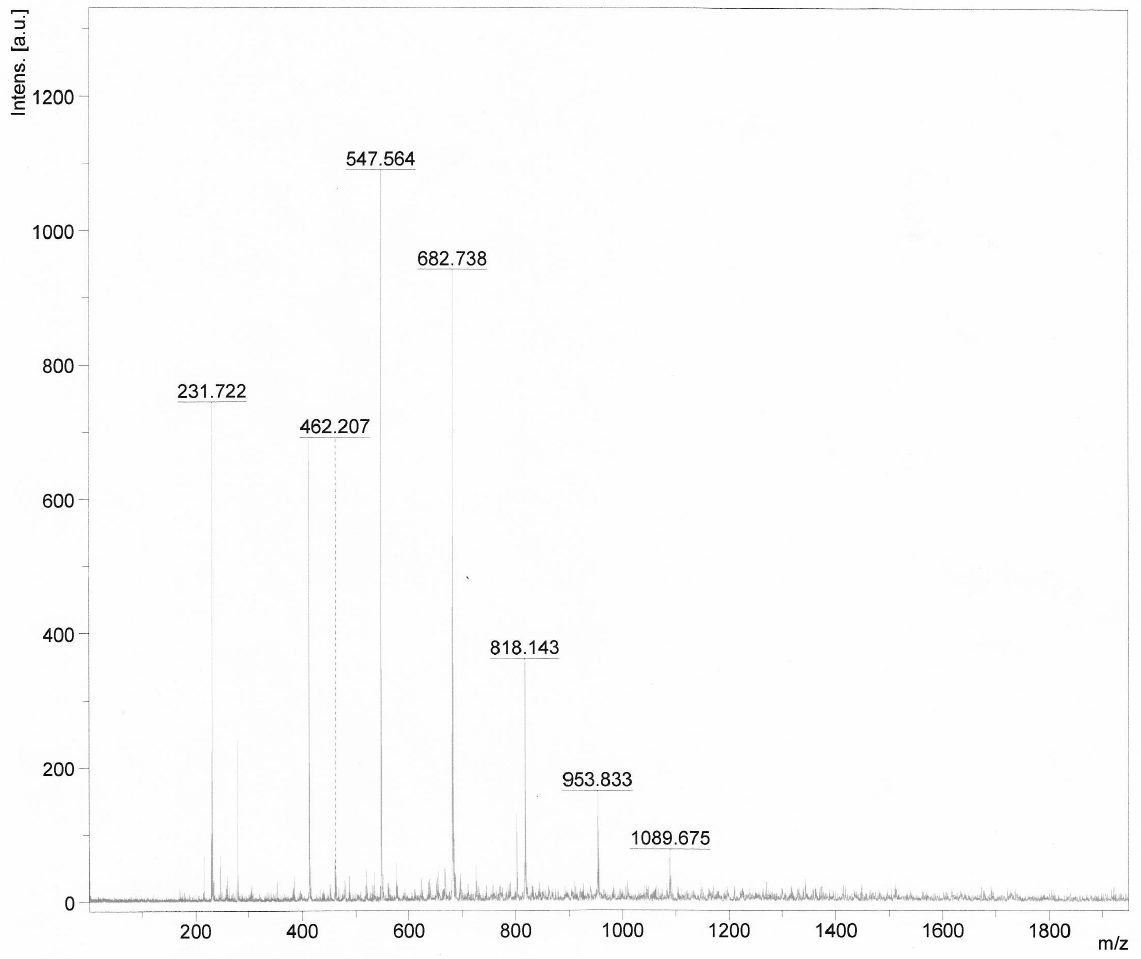


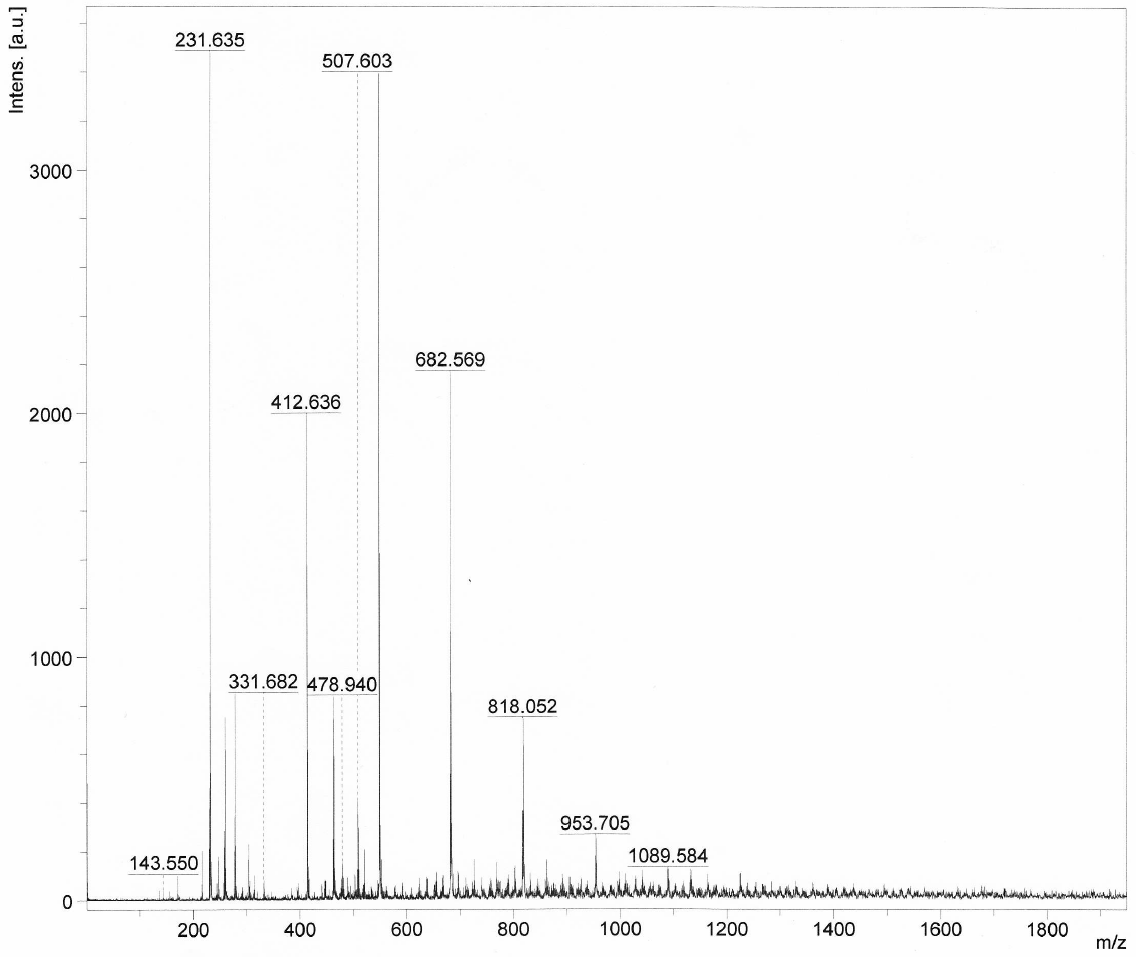


4,4',5,5'-tetramethoxy-2,2'-dimethyl-1,1'-biphenyl (**2a**)


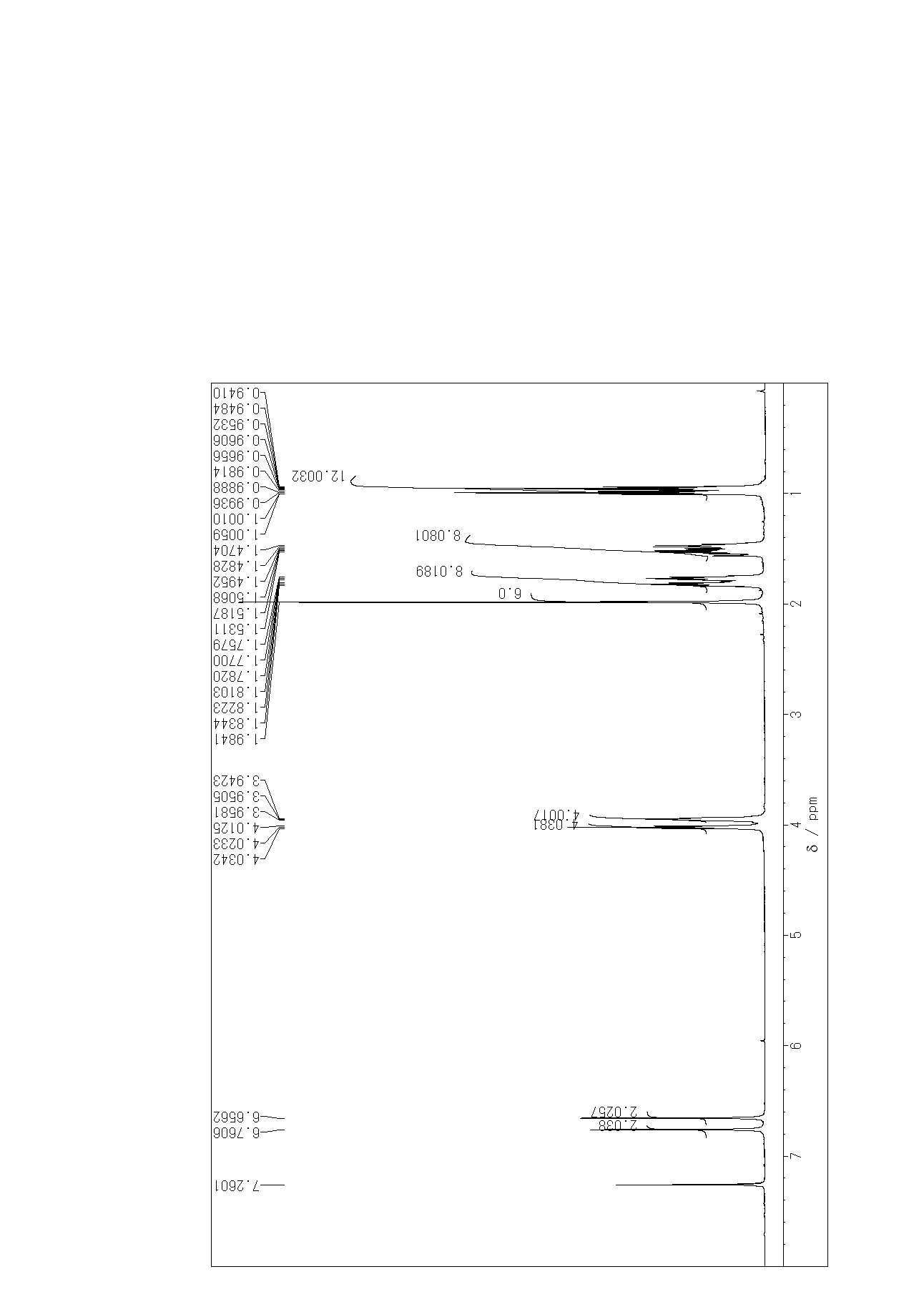


2,2'-difluoro-4,4',5,5'-tetramethoxy-1,1'-biphenyl (**2b**)


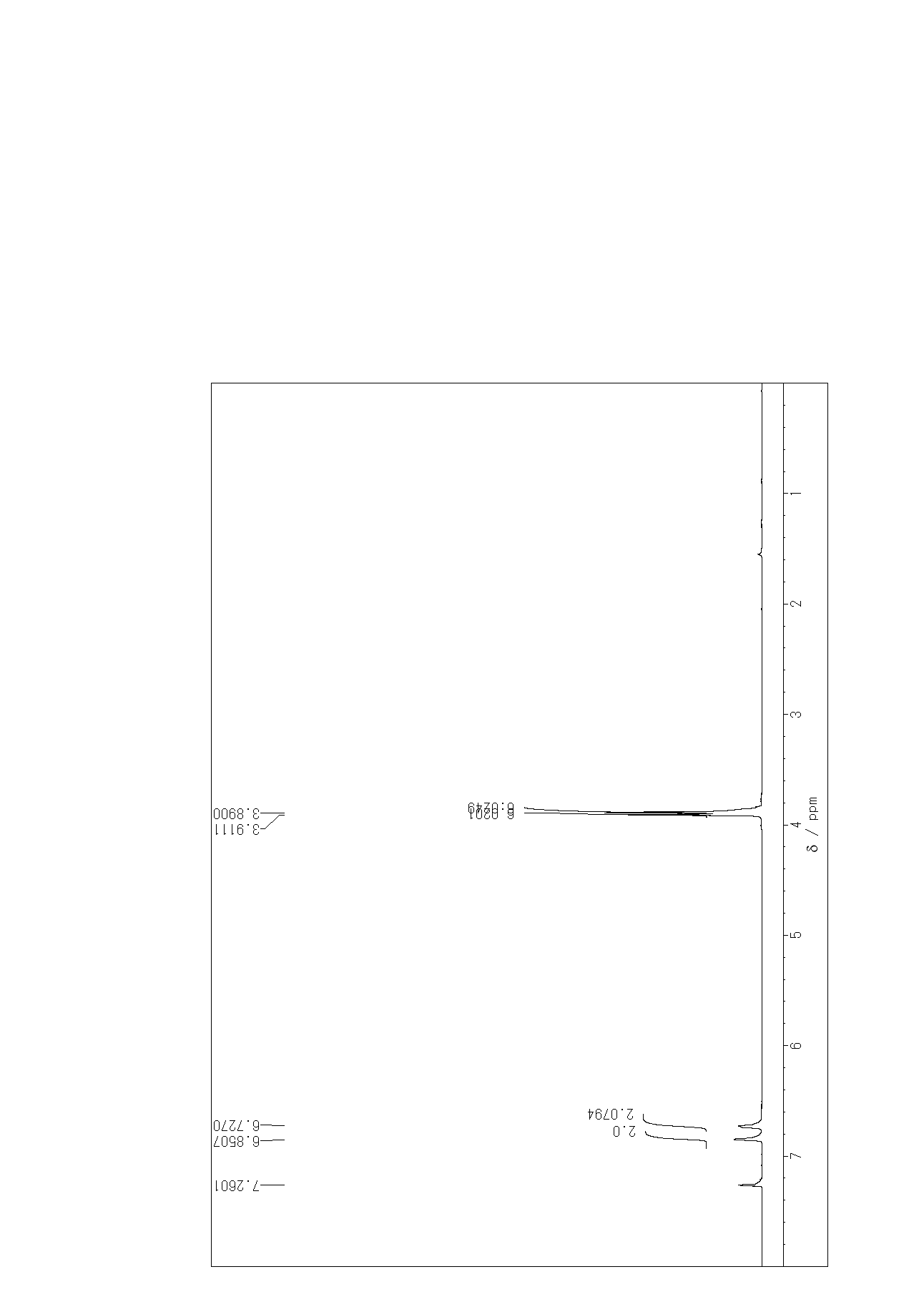


2,2'-difluoro-4,4',5,5'-tetramethoxy-1,1'-biphenyl (**2b**)


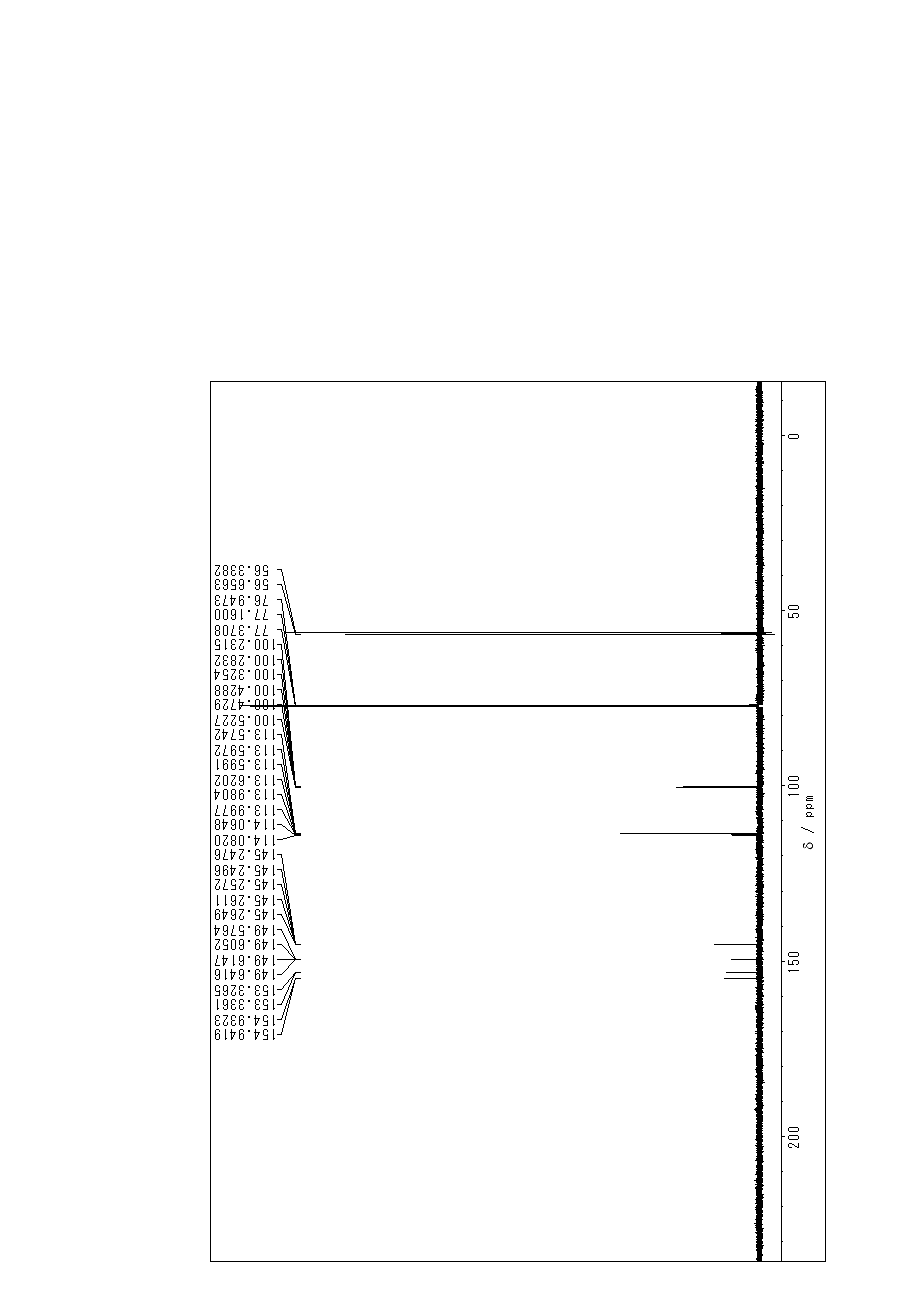


2,2'-dichloro-4,4',5,5'-tetramethoxy-1,1'-biphenyl (**2c**)


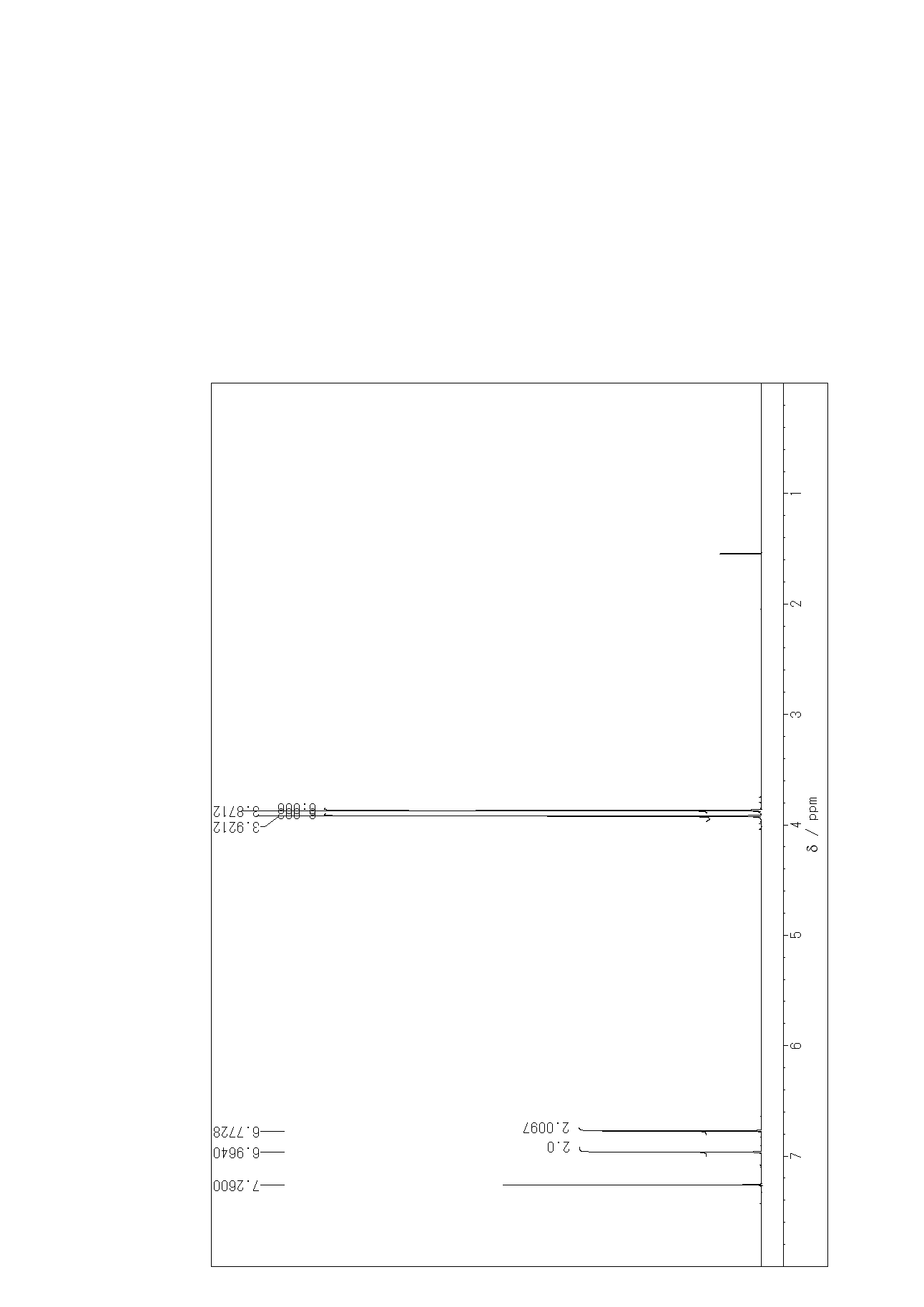


2,2'-dichloro-4,4',5,5'-tetramethoxy-1,1'-biphenyl (**2c**)


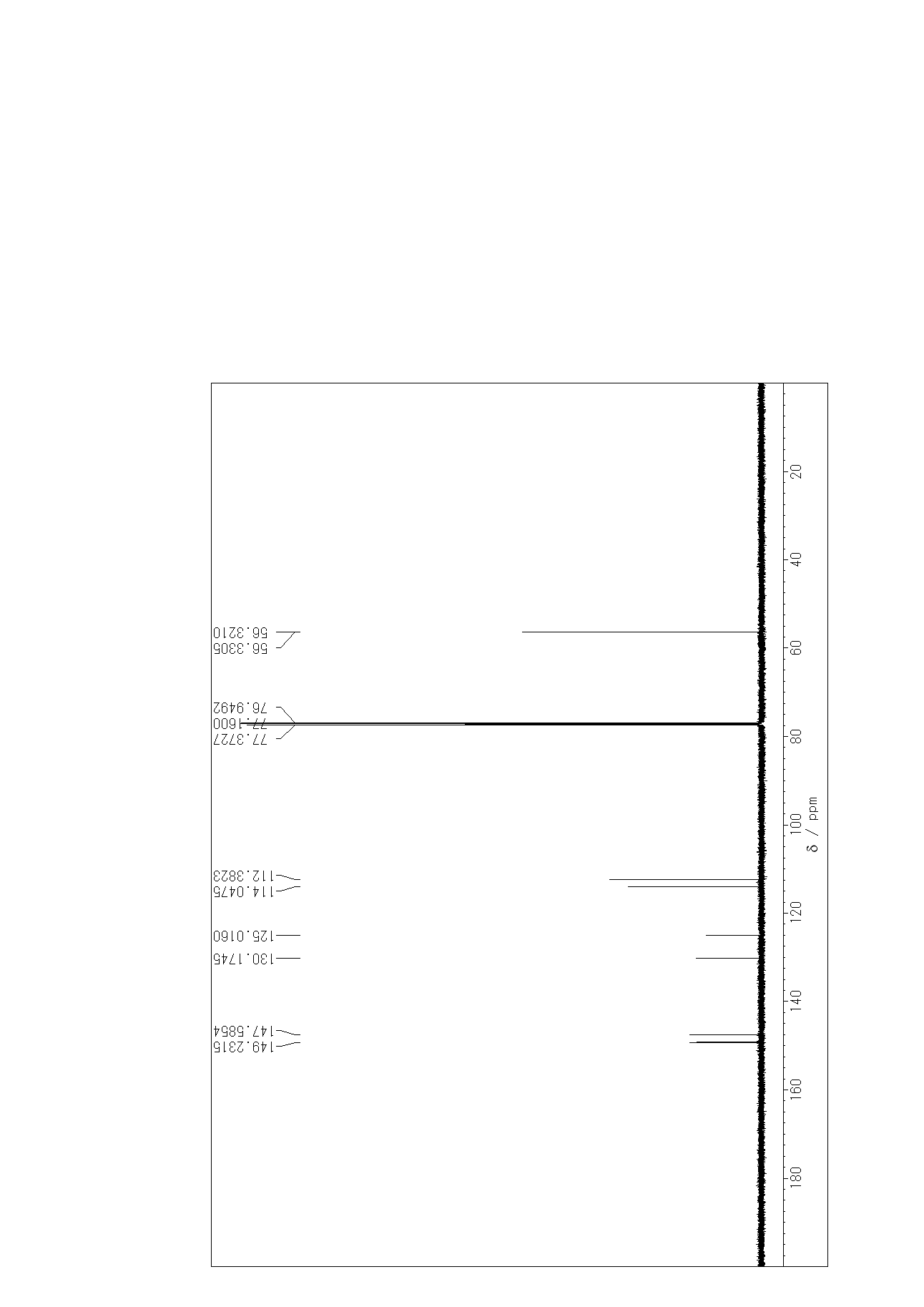


2,2'-dibromo-4,4',5,5'-tetramethoxy-1,1'-biphenyl (**2d**)


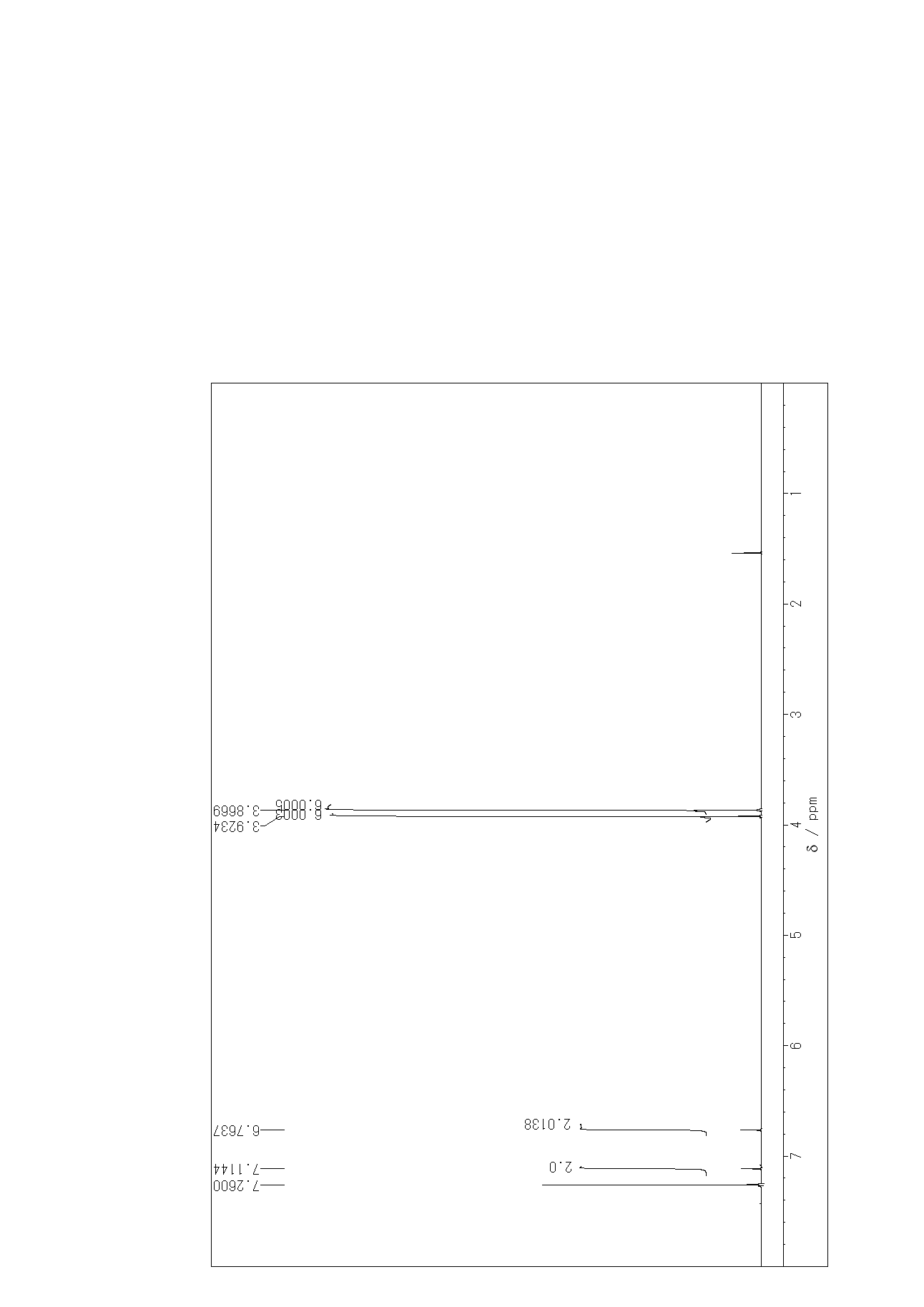


4,4',5,5'-tetrabuthoxy-2,2'-dimethyl-1,1'-biphenyl (**2g**)


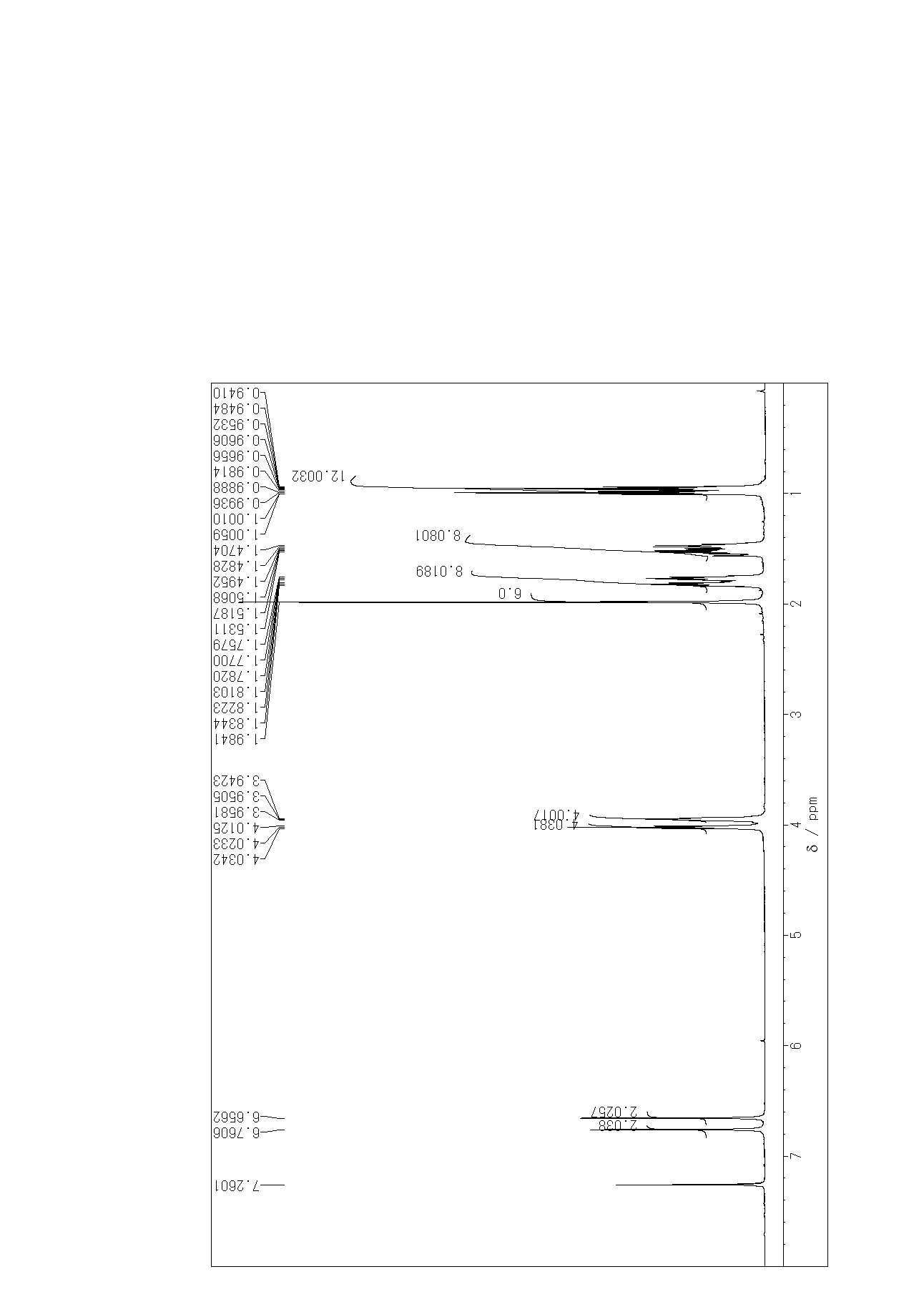


4,4',5,5'-tetrabuthoxy-2,2'-dimethyl-1,1'-biphenyl (**2g**)


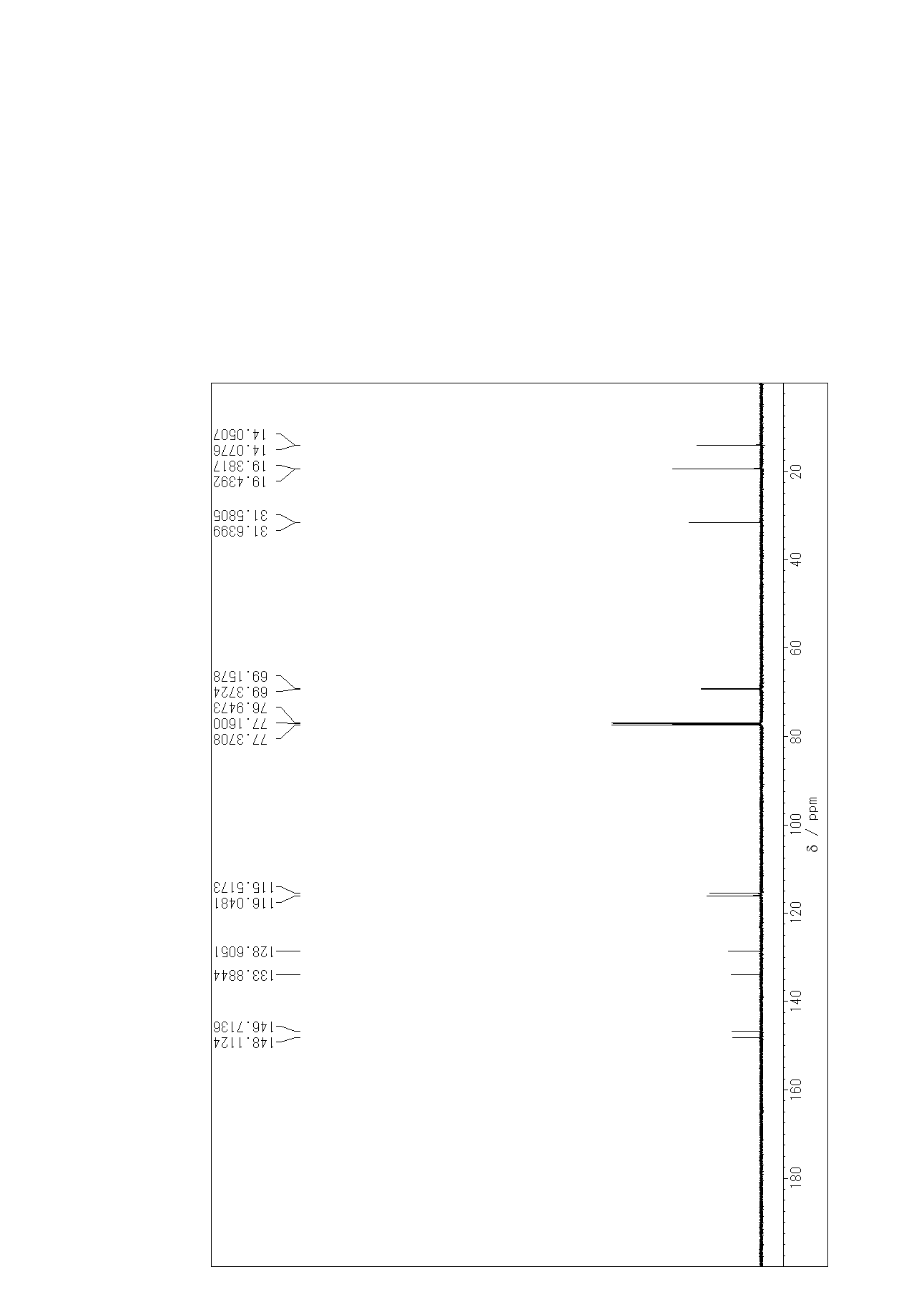


2,2',4,4',6,6'-hexamethoxy-1,1'-biphenyl (**4a**)


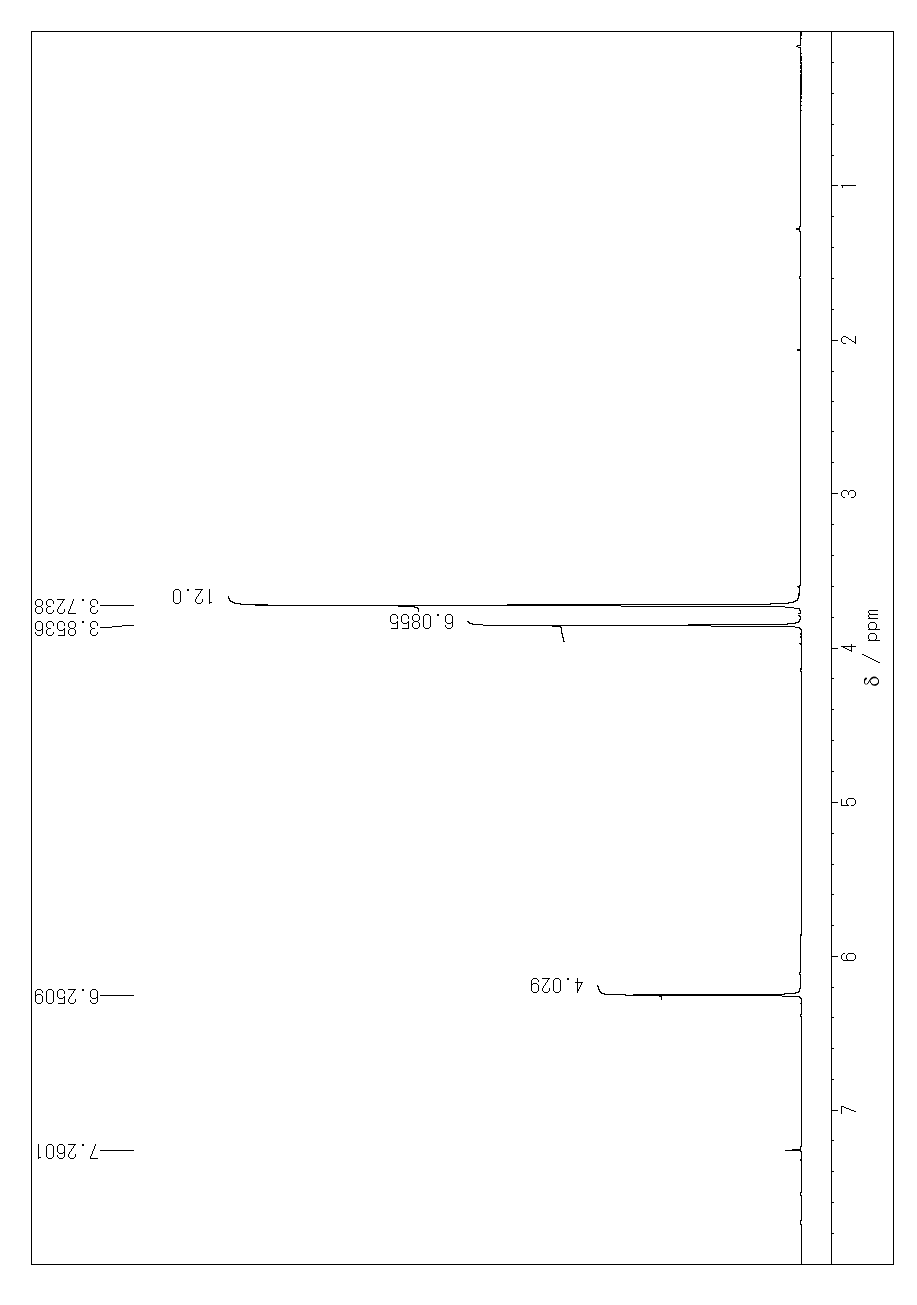


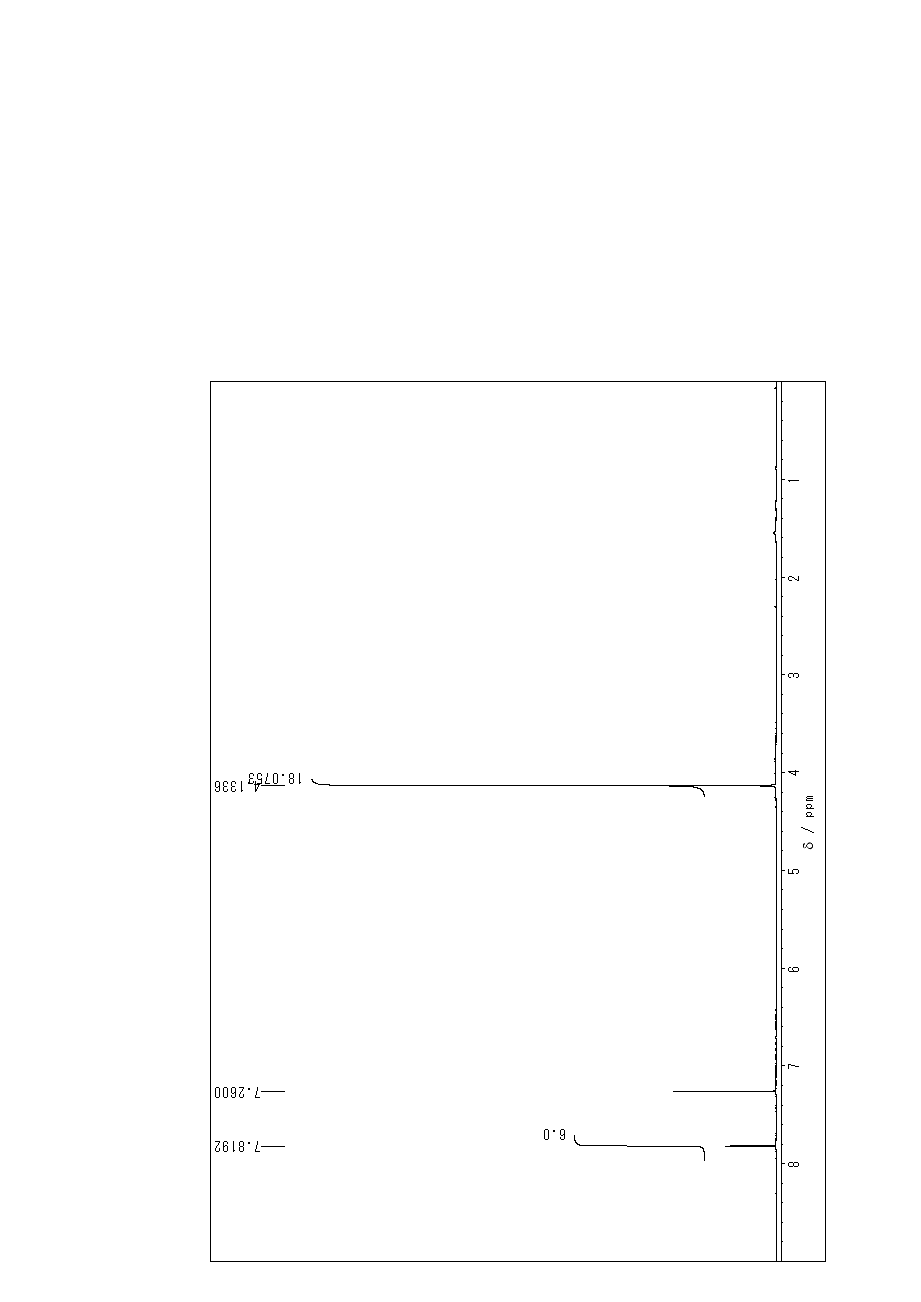
2,3,6,7,10,11-hexamethoxy triphenylene (**4b**)

2,2'-dimethoxy-1,1'-binaphthalene (**4e**)


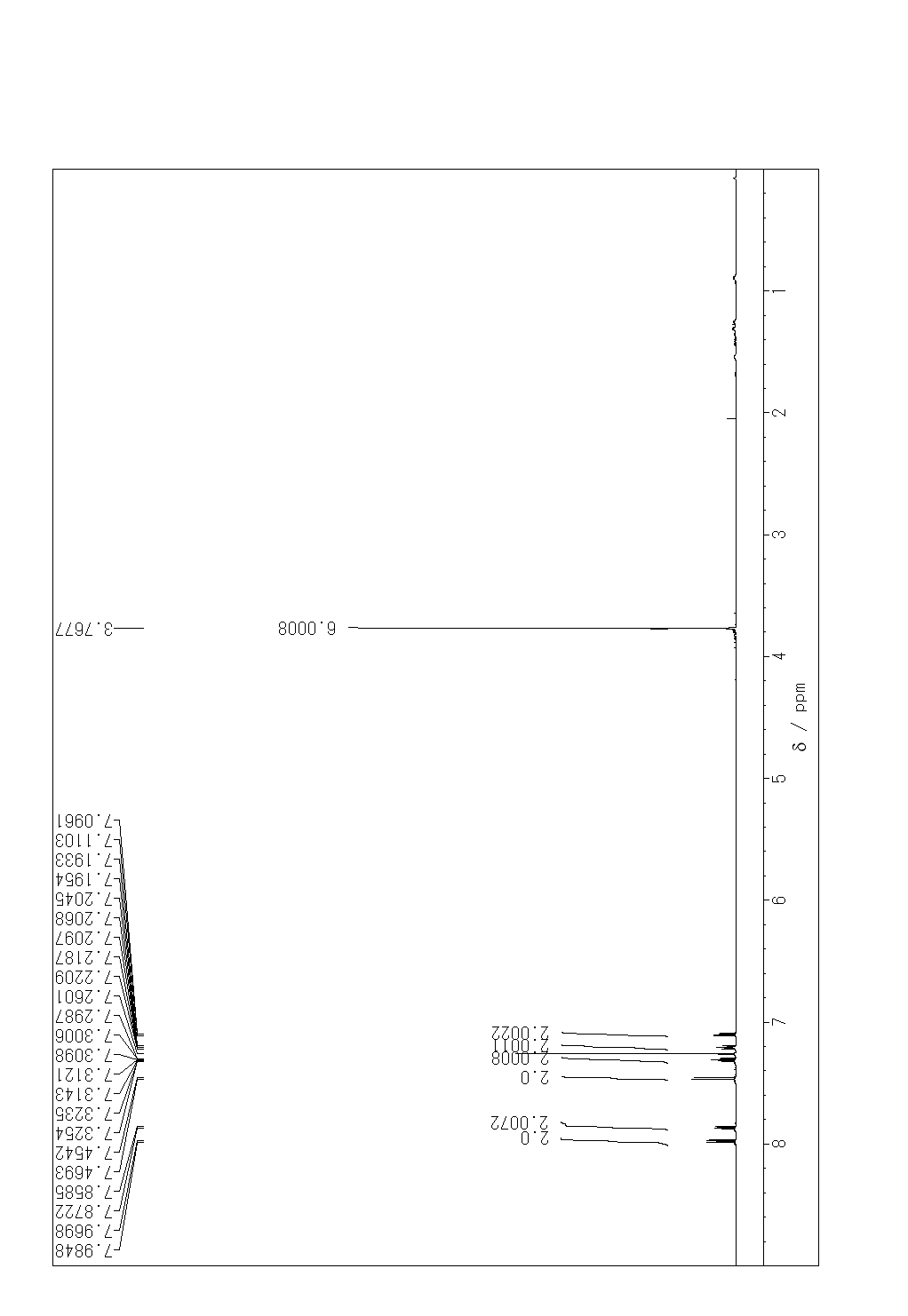


[

[1,1'-binaphthalene]-2,2'-diol (**4f**)


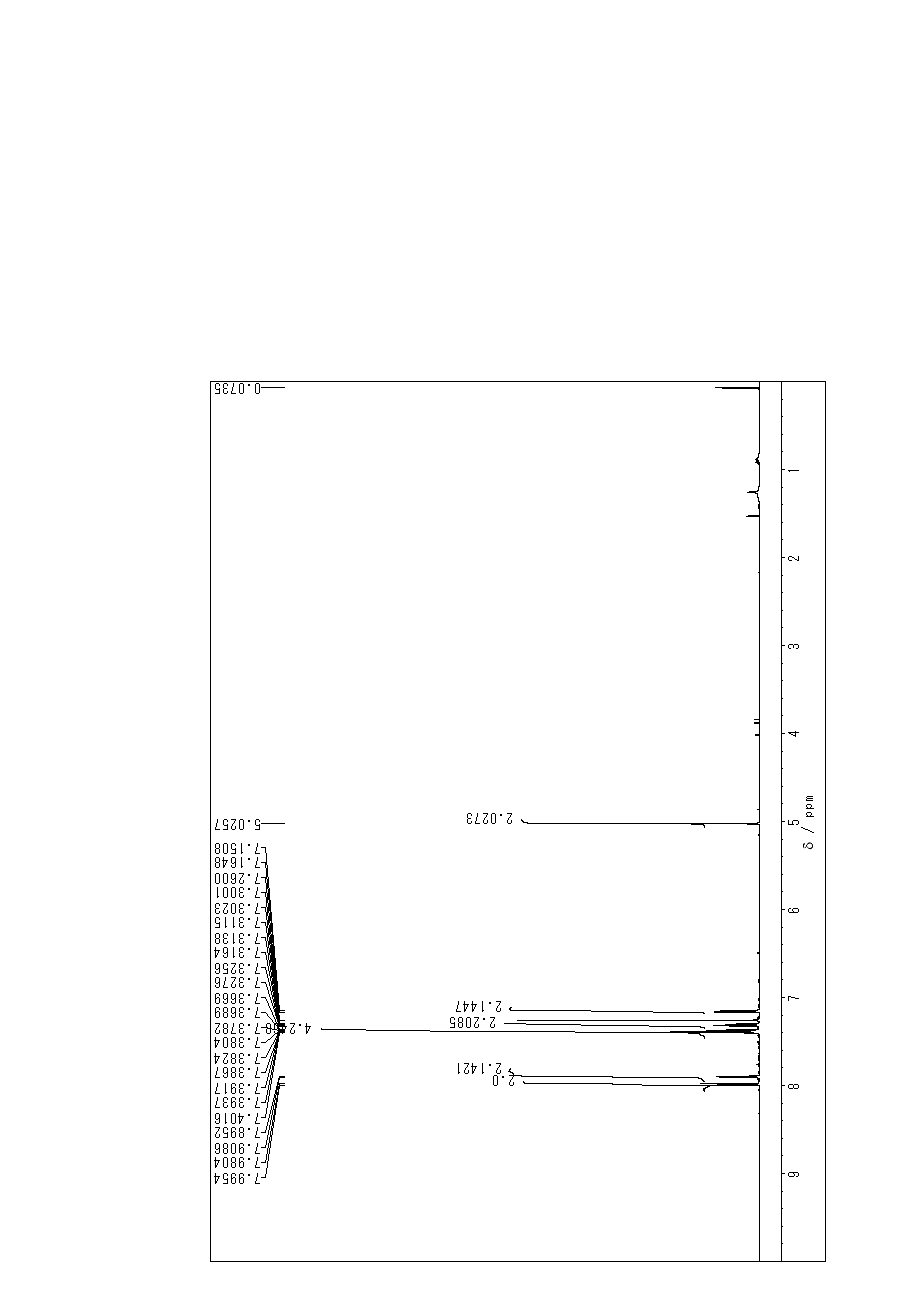


2,3,6,7-tetramethoxy triphenylene (**4g**)


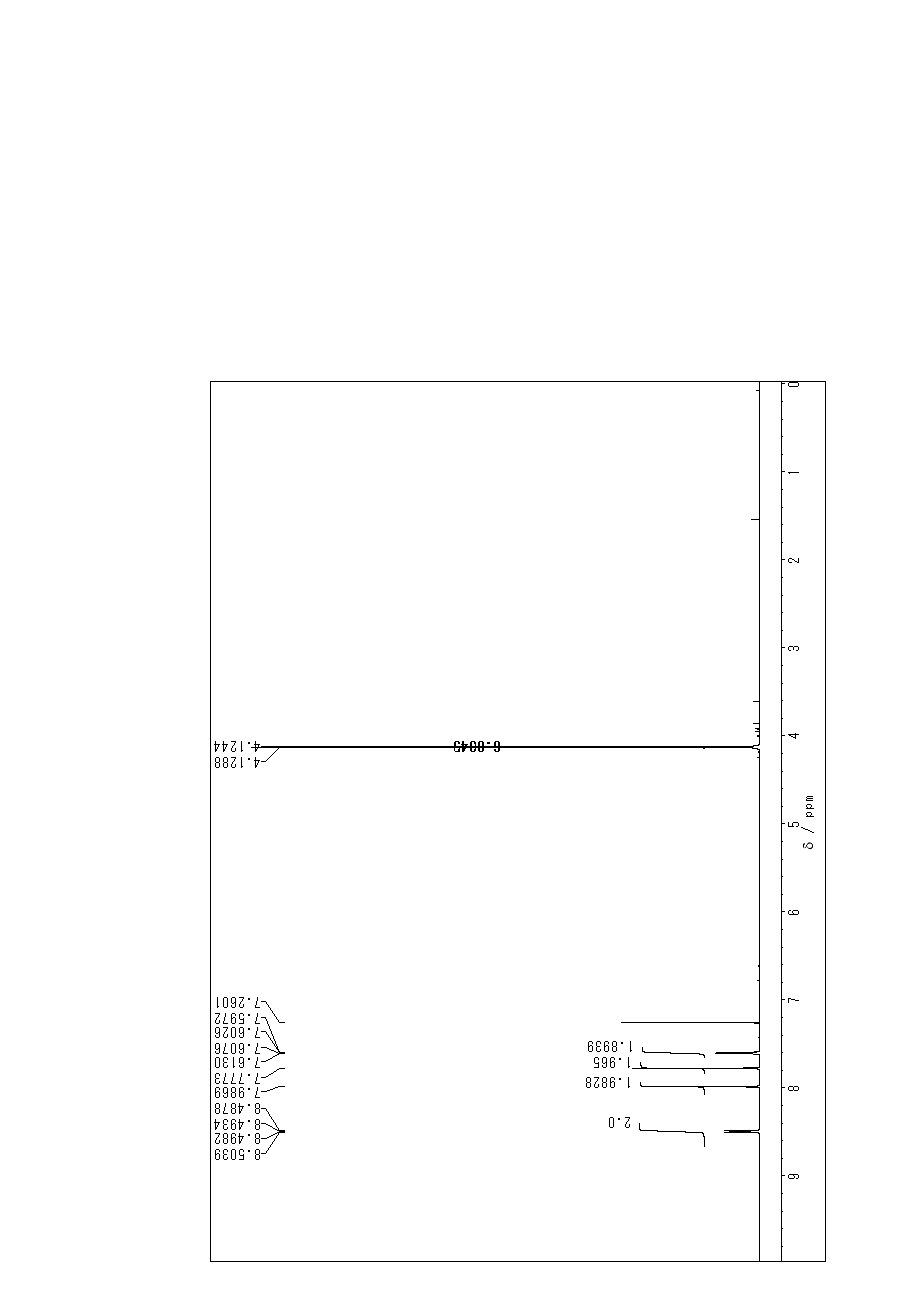


2,3,6,7-tetramethoxy phenanthrene (**4h**)


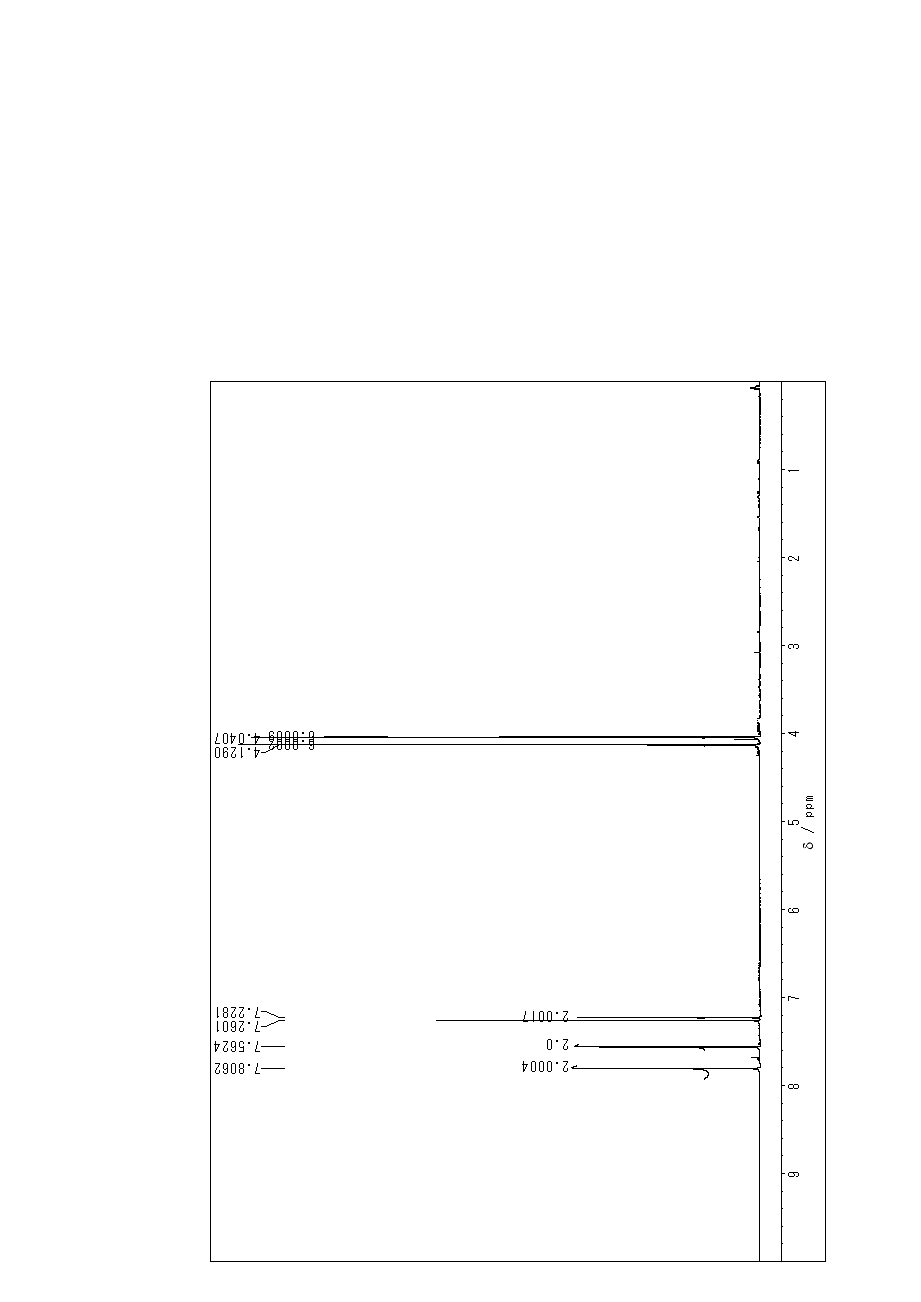


1-(2,4,6-trimethoxyphenyl)naphthalene (**4i**)


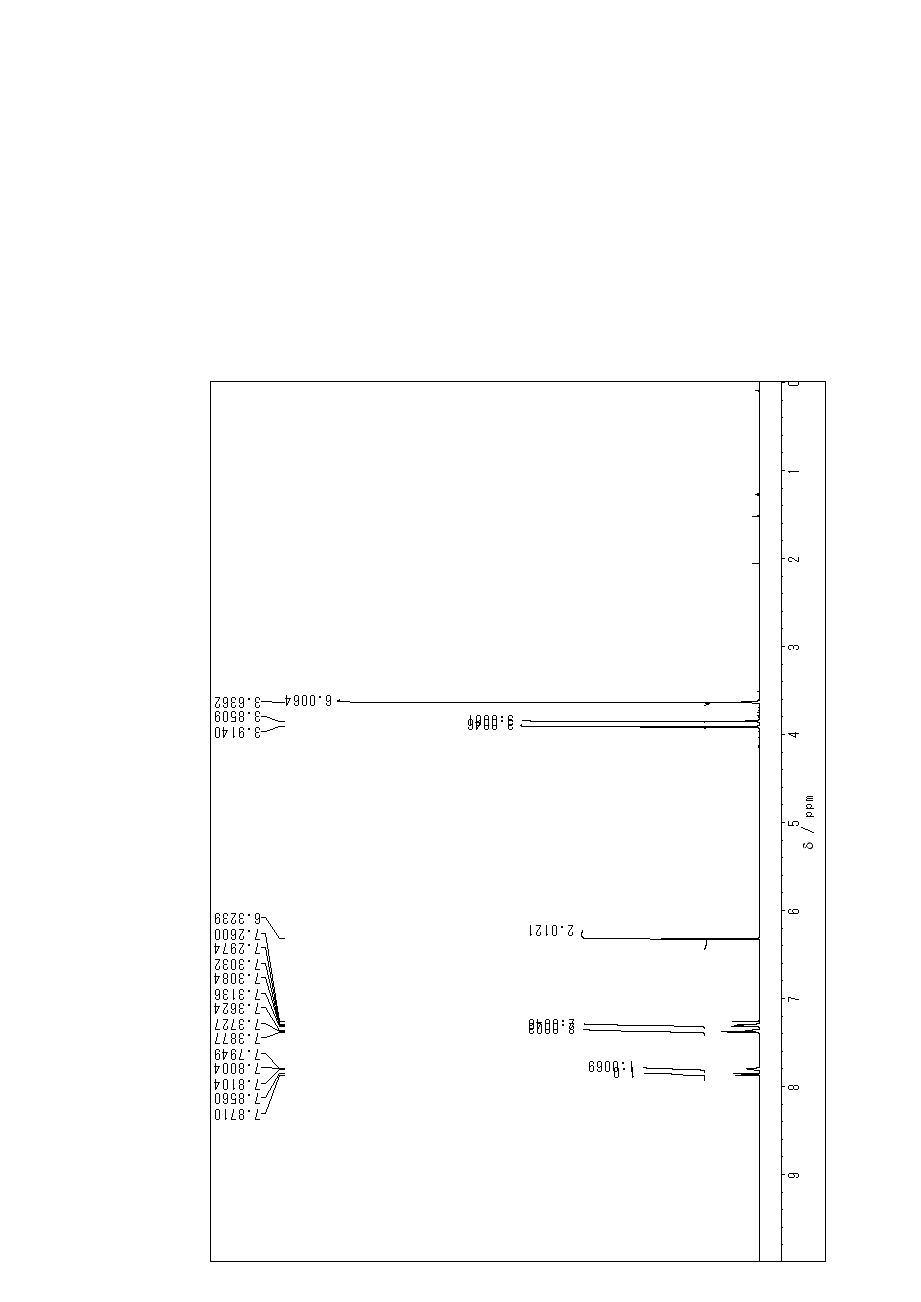


1-(2,4,6-trimethoxyphenyl)naphthalene (**4i**)


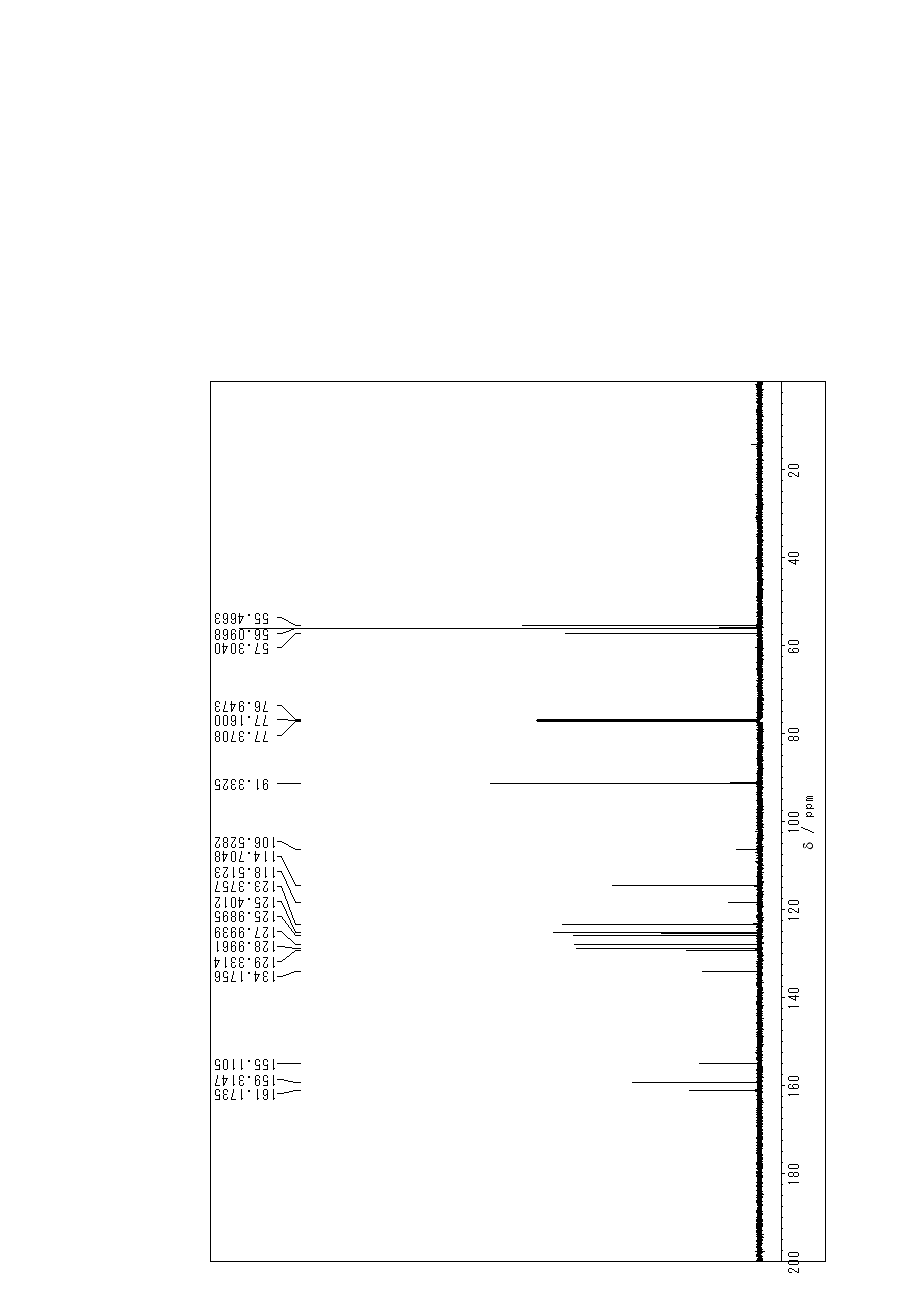


2-methoxy-1-(4,5-dimethoxy-2-methylphenyl)naphthalene (**4J)**


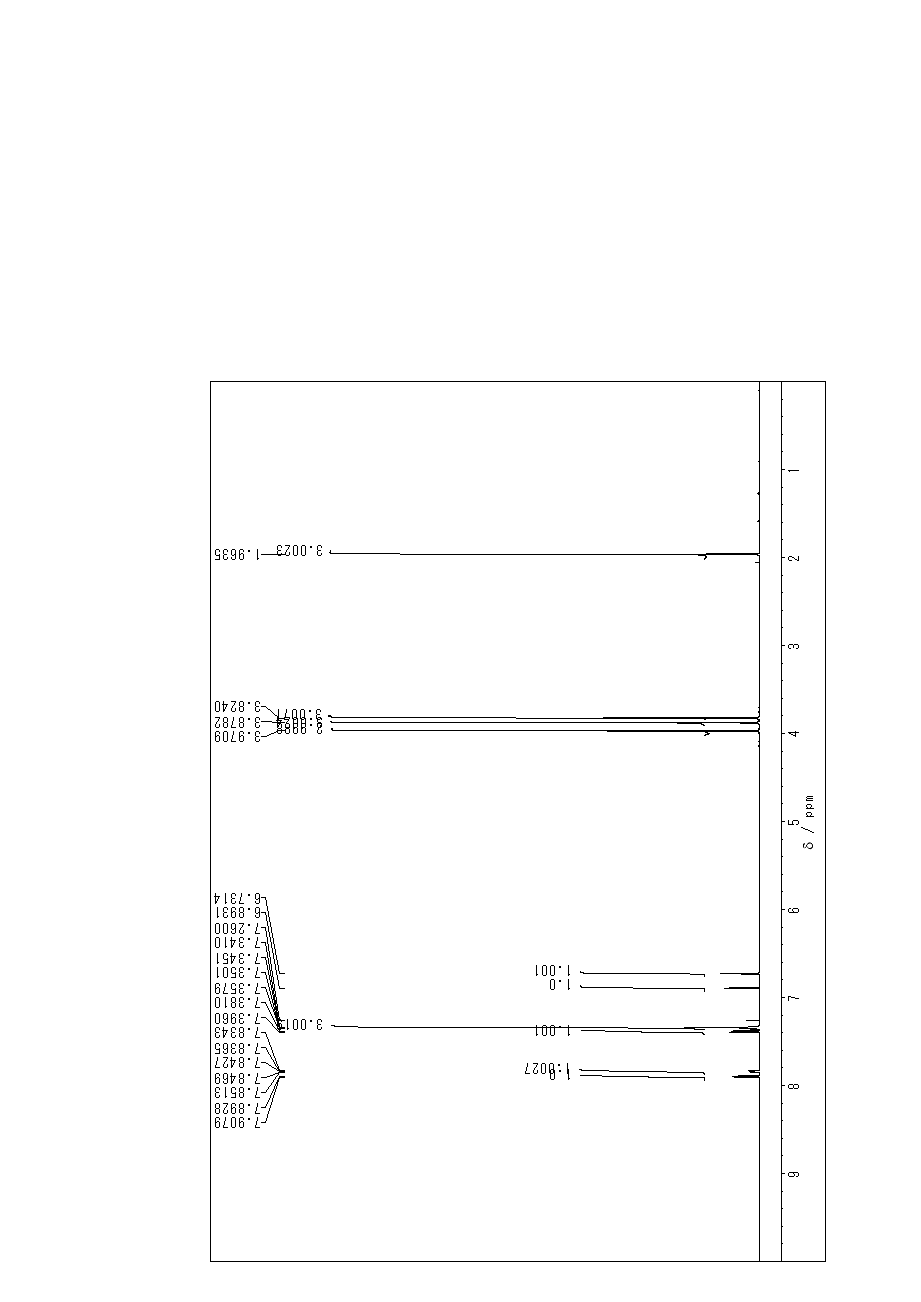


2-methoxy-1-(4,5-dimethoxy-2-methylphenyl)naphthalene (**4J)**


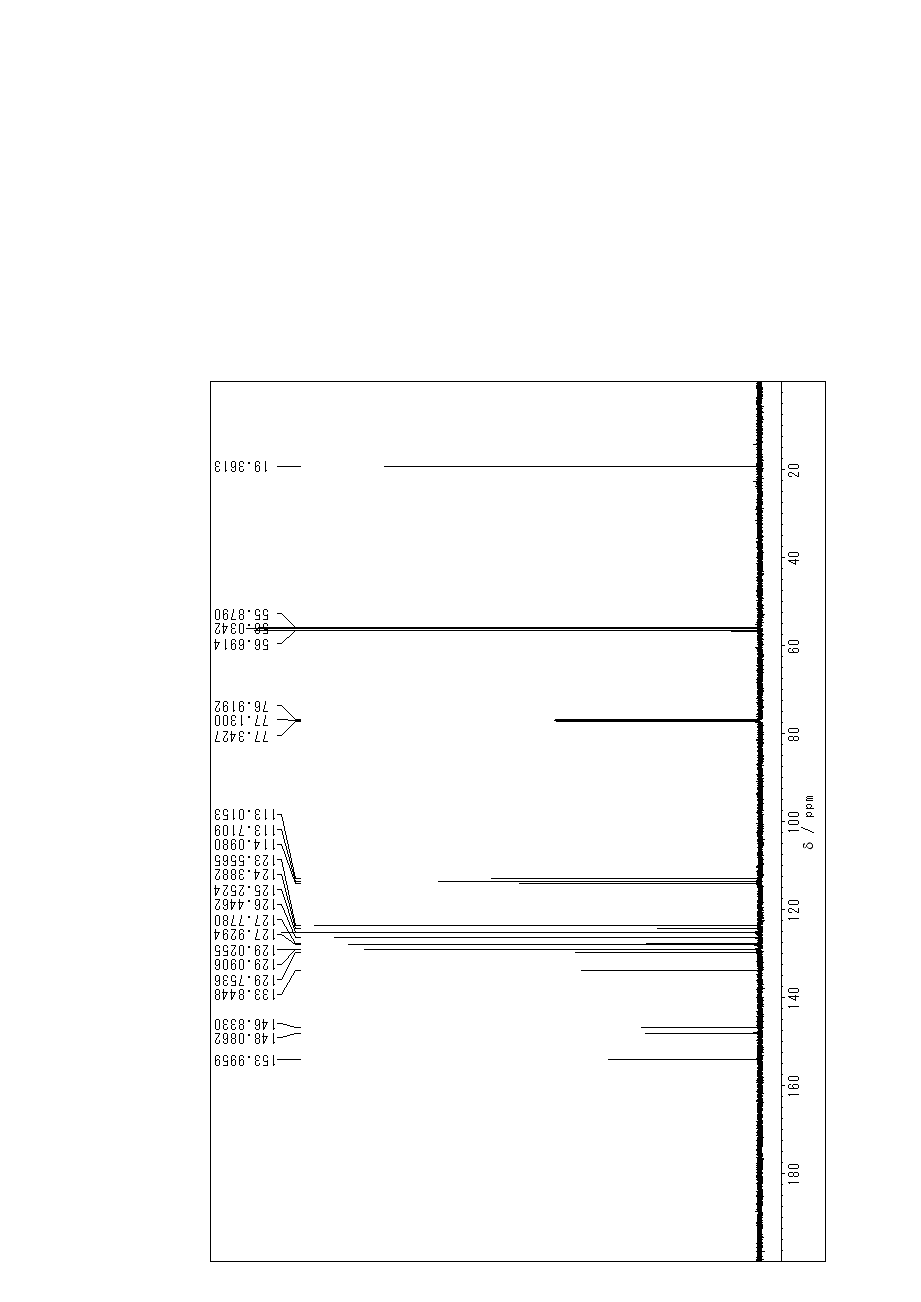


4-chloro-1,2-dimethoxybenzene (**1c**)


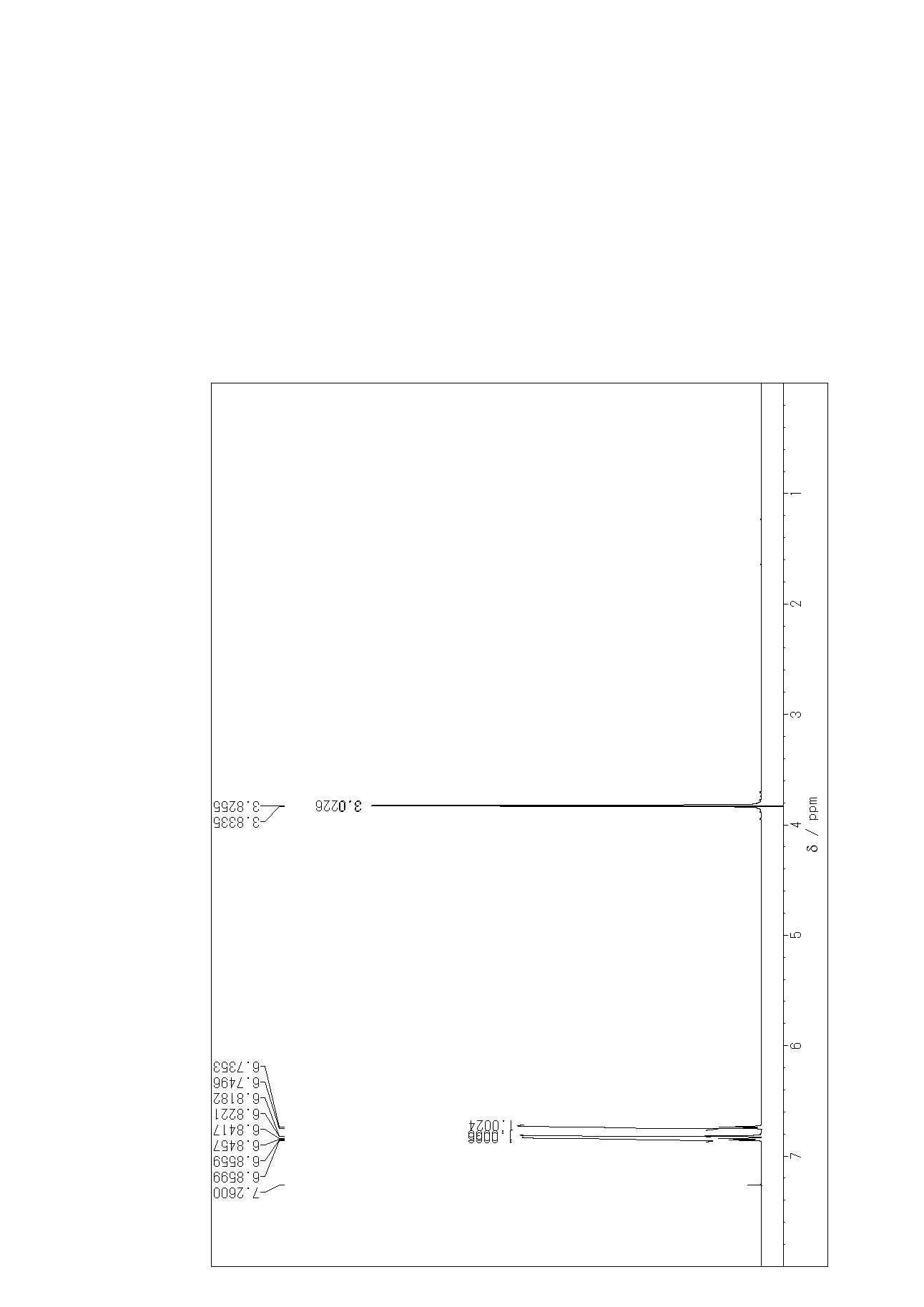


4-chloro-1,2-dimethoxybenzene (**1c**)

**
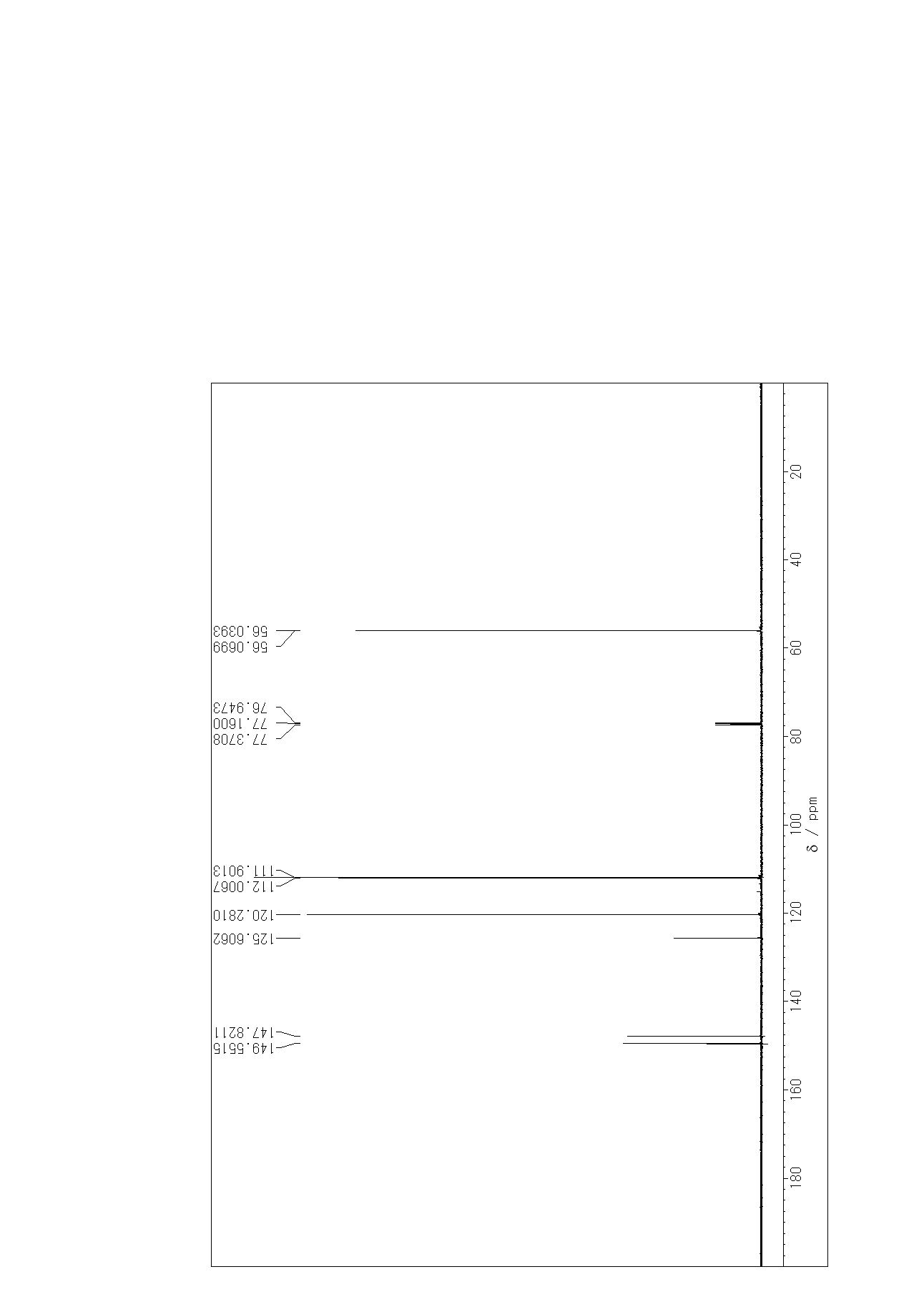
**


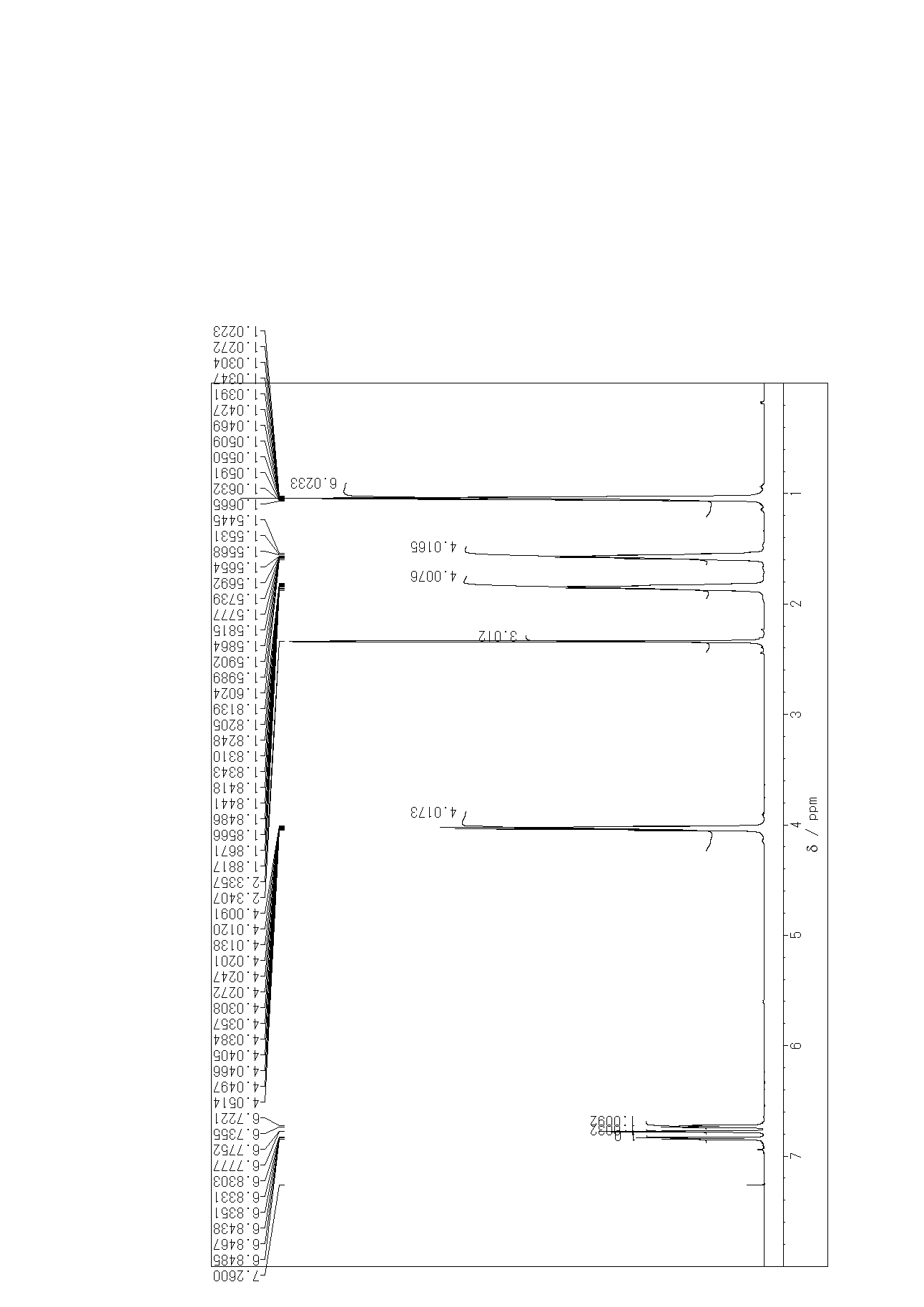
3,4-dibuthoxytoluene (**1g**)

3,4-dibuthoxytoluene (**1g**
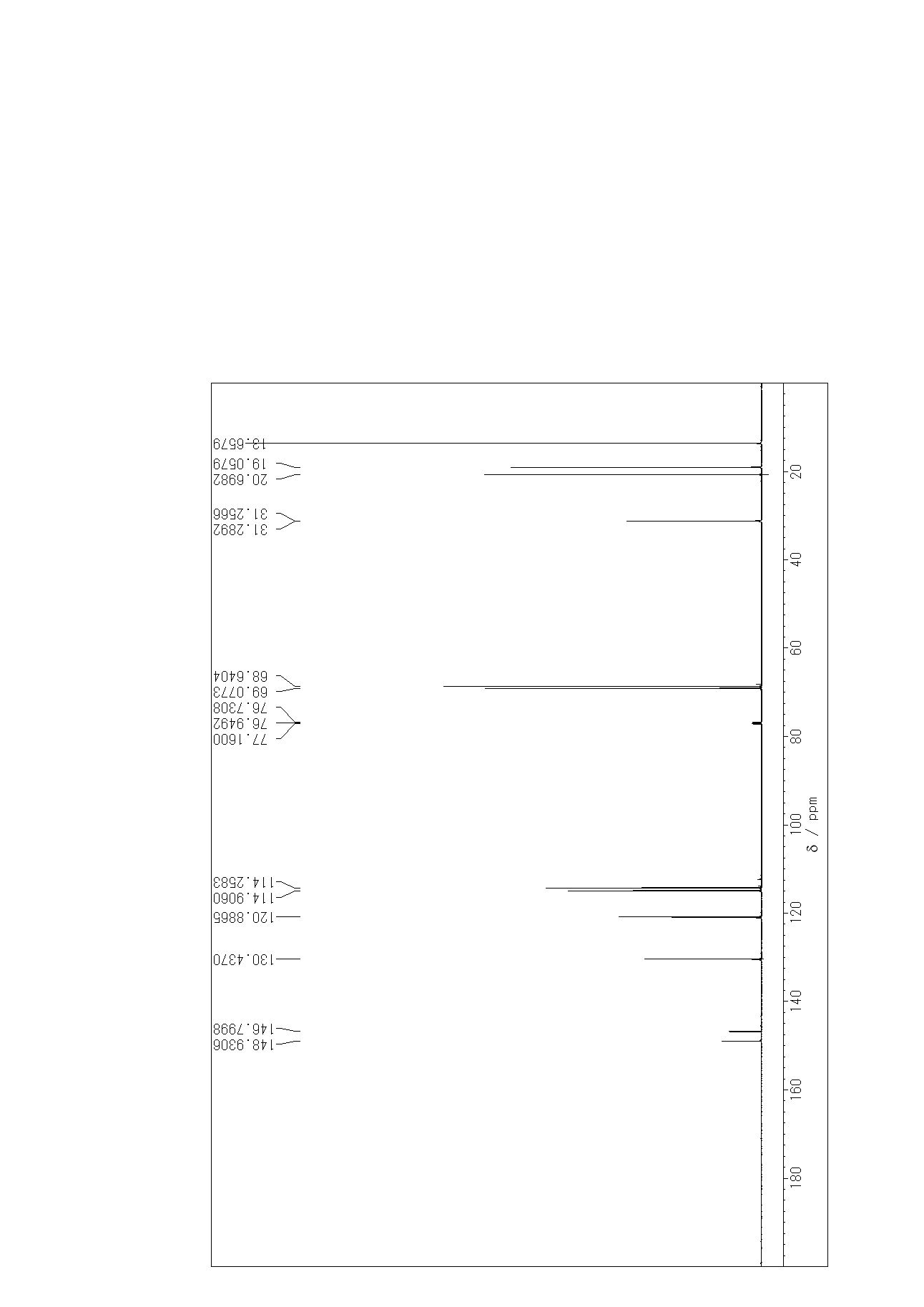
)

6-deuterio-3,4-dimethoxytoluene (**1aD**)


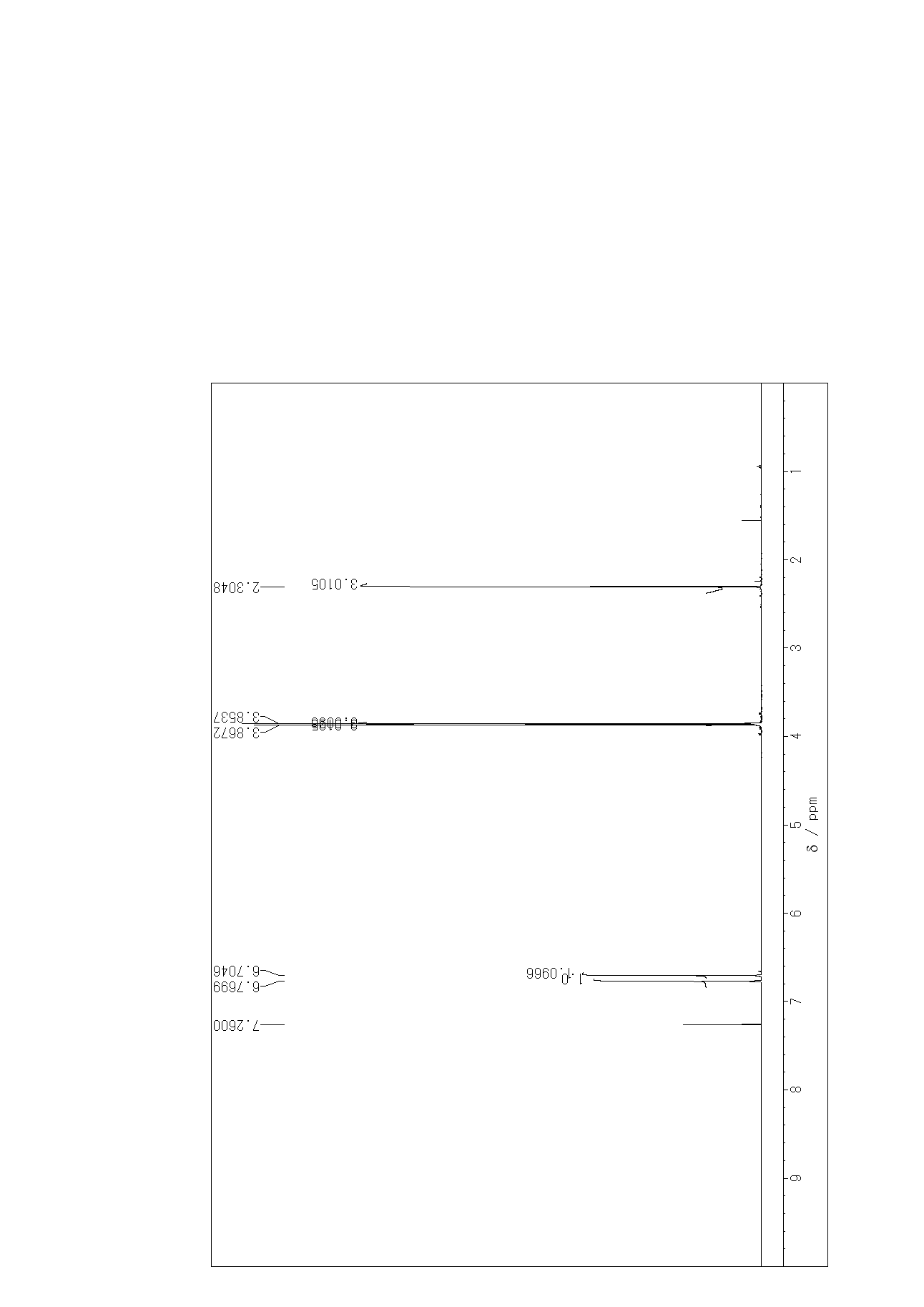


6-deuterio-3,4-dimethoxytoluene (**1aD**)

**
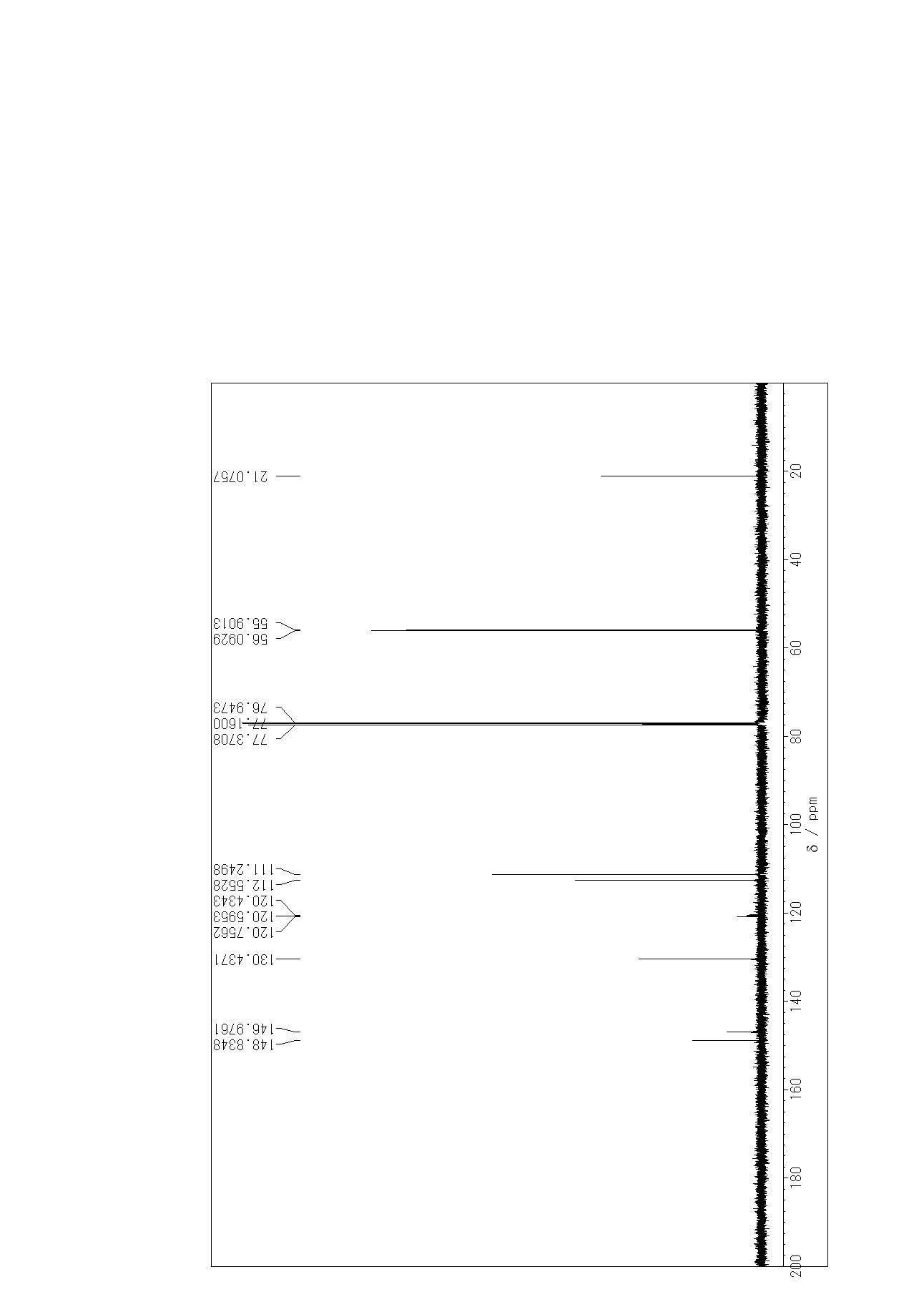
**

**23. Reference**

1. Jia, X., Wang, X., Xia, C. & Ding, K. Spiroketal-based phosphorus ligands for highly regioselective hydroformylation of terminal and internal olefins. *Chem. Eur. J.* **18**, 15288–15295 (2012).
2. Nose, M. & Suzuki, H. A Mild one-pot procedure for the polynitration of activated arenes. convenient preparation of dinitro- and trinitrodialkoxybenzenes. *Synthesis* **11**, 1539–1542 (2000).
3. Zhai, L., Shukla, R., Wadumethrige, S. H. & Rathore, R. Probing the arenium-Ion (protonTransfer) versus the cation-radical (electron transfer) mechanism of Scholl reaction using DDQ as oxidant. *J. Org. Chem.* **75,** 4748–4760 (2010).
4. Barrett, T. N., Braddock, D. C., Monta, A., Webb, M. R. & White, A. J. P. Total synthesis of the marine metabolite (±)-polysiphenol via highly regioselective intramolecular oxidative coupling. *J. Nat. Prod.* **74**, 1980–1984 (2011).
5. Tohma, H., Morioka, H. Takizawa, S., Arisawa M. & Kita Y. Effcient oxidative biaryl coupling reaction of phenol ether derivatives using hypervalent iodine(III) reagents. *Tetrahedron* **57**, 345–352 (2001).
6. Zhai, L., Shukla, R. & Rathore, R. Oxidative C–C bond formation (Scholl reaction) with DDQ as an efficient and easily recyclable oxidant. *Org. Lett.* **11**, 3474–3477 (2009).
7. Navajas, P. M., Asenjo, N. G., Santamaria, R., Menéndez, R., Corma, A. & García, H., *Langmuir* **29**, 13443–13448 (2013).
